# NiO-Microflower Formed by Nanowire-weaving Nanosheets with Interconnected Ni-network Decoration as Supercapacitor Electrode

**DOI:** 10.1038/srep11919

**Published:** 2015-07-13

**Authors:** Suqing Ci, Zhenhai Wen, Yuanyuan Qian, Shun Mao, Shumao Cui, Junhong Chen

**Affiliations:** 1 Department of Mechanical Engineering, University of Wisconsin-Milwaukee, Milwaukee Wisconsin 53211, United States; 2 Key Laboratory of Jiangxi Province for Persistent Pollutants Control and Resources Recycle, Nanchang Hangkong University, Nanchang 330063, PR China

## Abstract

We propose a ‘weaving’ evolution mechanism, by systematically investigating the products obtained in controlled experiments, to demonstrate the formation of Ni-based ‘microflowers’ which consists of multiple characteristic dimensions, in which the three dimensional (3D) NiO ‘microflower’ is constructed by a two-dimensional (2D) nanosheet framework that is derived from weaving one-dimensional (1D) nanowires. We found such unique nanostructures are conducive for the generation of an electrically conductive Ni-network on the nanosheet surface after being exposed to a reducing atmosphere. Our study offers a promising strategy to address the intrinsic issue of poor electrical conductivity for NiO-based materials with significant enhancement of utilization of NiO active materials, leading to a remarkable improvement in the performance of the Ni-NiO microflower based supercapacitor. The optimized Ni-NiO microflower material showed a mass specific capacitance of 1,828 F g^−1^, and an energy density of 15.9 Wh kg^−1^ at a current density of 0.5 A g^−1^. This research not only contributes to understanding the formation mechanism of such ‘microflower’ structures but also offers a promising route to advance NiO based supercapacitor given their ease of synthesis, low cost, and long-term stability.

There is current interest in the design and manufacture of various two-dimensional (2D) ultrathin layered nanosheet materials^1^,^2^. 2D nanostructures may find use in more efficient energy-producing, energy-absorbing, and energy storage devices, because their ultrahigh fraction of surface atoms could enable nearly full use of active materials^3^,^4^,^5^,^6^,^7^,^8^,^9^. 2D nanomaterials can be prepared through either a “bottom-up” approach^10^, including assembly of nanoclusters^11^,^12^, coalescence of nanowires^13^,^14^, and oriented attachment of nanocrystals^15^,^16^, or a “top-down” method, e.g., exfoliation of layer-structured bulk materials^17^,^18^,^19^,^20^. Both “bottom-up” and “top-down” synthesis techniques usually involve the use of organic solvents, polymeric stabilizers, surface capping agents, and burdensome post-processing. Moreover, there is still a lack of effective methods to integrate 2D nanosheets into three-dimensional (3D) hierarchical macroscopic structures with active materials at exposed/available surfaces in the open-framework 3D structure^21^,^22^.

Nickel oxide (NiO) has been recognized as one of the most promising electrode materials for pseudocapacitor applications in aqueous alkaline electrolytes due to its high theoretical specific capacitance (2,584 F g^−1^ within 0.5 V), low cost, and excellent chemical and thermal stability^23^,^24^,^25^,^26^,^27^,^28^,^29^,^30^,^31^. However, the poor electrical conductivity of NiO increases both the sheet resistance and the charge transfer resistance of the electrode, which in work reported to date has led to a capacitance value much less than the theoretical value and a poor rate capability^29^,^30^. Consequently, various methods, such as modification with nanostructured carbon^24^,^25^,^26^, preparation of Ni-NiO nanocomposites^27^,^28^,^29^,^30^,^31^, and synthesis of nanostructures with a unique morphology^32^,^33^,^34^,^35^,^36^, have been proposed to improve the NiO supercapacitor performance. In recent years, there have been numerous reports on preparing hierarchical NiO structures that are promising for energy storage systems because of their extraordinary properties, such as high surface area and unique geometrical structure^37^,^38^,^39^,^40^,^41^,^42^,^43^. Despite the advancements, very little research has been done to investigate the formation mechanism of such 3D hierarchical structures, and extensive research is still required to explore effective strategies for achieving high-efficiency utilization of NiO with further performance improvement.

We herein present a systematic research to the formation of a hierarchical NiO nanostructure with multiple characteristic dimensions, and propose a ‘weaving’ mechanism for the formation based on abundant experiments. The 3D NiO ‘microflowers’ are constructed of 2D ultrathin nanosheets that can be visualized as “woven” from one-dimensional (1D) nanowires. Benefiting from such a unique structure, an interconnected nickel (Ni) conductive network can be generated *in situ* on the surface of the NiO nanosheets through a controllable reduction process. The resulting Ni-NiO hybrid has an open hierarchical 3D structure and good electrical conductivity, as shown in Fig. 1, which offers great potential for supercapacitor applications, including easy transport of ions/electrolytes at the interface of the electrode/electrolyte, fast electron transfer on the surface of the nanosheets, and high-efficiency use of the active material NiO.

## Results

### Synthesis

The overall method is described in in the supplementary material (Supplementary, Fig. S1). Briefly, the precursor was prepared by dissolving sodium dodecyl sulphate (SDS), urea, and NiCl_2_ in water. The mixed solution was then transferred to an autoclave for hydrothermal treatment. Several parameters, such as the proportion of reactants, temperature, and reaction time, were examined which in turn made it possible to explore the formation mechanisms of the corresponding nanostructures. In our synthesis process, only urea and SDS were used as auxiliary reagents to grow Ni-based nanostructures. The hydrolysis of urea led to the generation of OH^−^ and CO_3_^2−^ which then reacted with Ni^2+^ to form a green NiCO_3_∙Ni(OH)_2_∙xH_2_O precipitate (Supplementary, Fig. S1b and Fig. S2a). The resulting precipitate was subsequently decomposed to a black NiO powder upon annealing (Supplementary, Fig. S1c and Fig. S2b), as proven by thermogravimetric analysis (TGA). The NiO sample was then partially reduced to produce a grey Ni-NiO sample (Supplementary, Fig. S1d) under an H_2_/Ar atmosphere.

### Microflowers constructed from Ni nanosheets

When no SDS was involved in the reactions, the morphology of the samples was strongly influenced by the molar ratio of NiCl_2_/urea. Porous Ni-based nanoplates were formed with a high molar ratio NiCl_2_:urea = 5:1 (Supplementary, Fig. S3a, b); and microspheres constructed from nanowires and visually similar to silkworm cocoons were produced with a molar ratio NiCl_2_:urea = 3:1 (Supplementary, Fig. S3c, d); while a porous microsphere with well-defined nanowire building blocks was obtained for a molar ratio NiCl_2_:urea = 1:1 (Supplementary, Fig. S3e, f). For NiCl_2_:urea = 1:2, the microsphere size decreased and the nanowires became less discernible, possibly due to the high agglomeration of nanowires (Supplementary, Fig. S4a, b). When the molar ratio was decreased to NiCl_2_:urea = 1:10, the ‘microspheres’ were deformed (Supplementary, Fig. S4c, d). For no urea and excess urea (e.g., a molar ratio NiCl_2_:urea = 1:30), there was no solid product formed.

**Figure 2:**
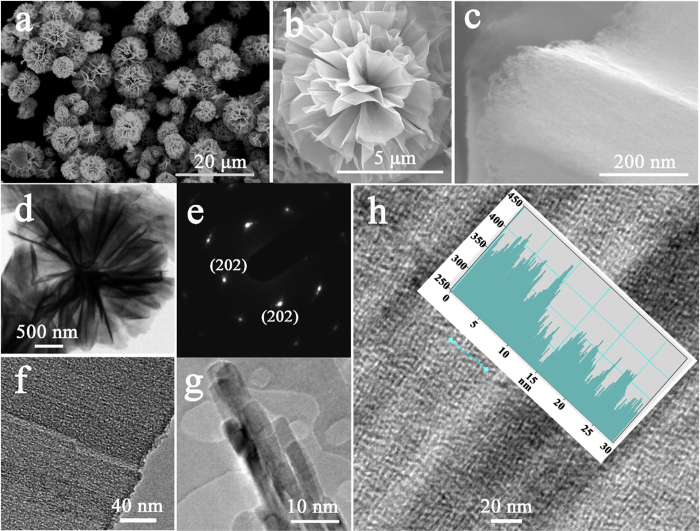
(**a**–**c**) SEM images of NiO microflowers at different magnifications; (**d**)TEM image of NiO microflowers; (**e**) SAED pattern of NiO nanosheets in a microflower; (**f**–**h**) Magnified TEM images of nanosheets in a NiO microflower; inset of (**h**) is the line scan intensity profile corresponding with the cyan line. (Note: the amount of NiCl_2_ and urea was fixed at 5 mmol, the amount of SDS was 0.6 g, and the reaction temperature was 150 °C).

The influence of the concentration of SDS on the product morphology was also studied with both NiCl_2_ and urea fixed at 5 mmol. For 0.1 g SDS, two types of nanostructures formed (Supplementary, Fig. S5a-c): one was nanowire-constructed porous microspheres (Fig. S5b) that are similar to the products (Fig. S3e, f) obtained without SDS, and the other was ‘microflowers’ composed of nanosheets that were evidently formed from “woven” nanowires (Fig. S5c). For 0.3 g SDS, the samples also contained nanowire-constructed porous microspheres (Fig. S5e) and nanosheet-constructed ‘microflowers’ (Fig. S5f), but a larger fraction of microflowers (Supplementary, Fig. S5d) was observed compared with the samples obtained with 0.1 g SDS. Notably, for 0.6 g SDS added into the reaction system, only microflowers were obtained as shown in Fig. 2, which presents the morphology and the crystalline structure of the as-prepared NiO microflowers. Figure 2a shows a typical scanning electron microscope (SEM) image, confirming that the samples are actually flower-like structures with a size range of 1–10 μm. Figure 2b shows an SEM image of one flower-like part, in which the entire 3D structure is built from a large number of nanosheets. Figure 2c shows the ridge surface on a single nanosheet, in which the ‘smooth’ surface looks like a densely woven gauze. Figure 2d shows a typical transmission electron microscope (TEM) image of one NiO microflower, again illustrating that the products have a 3D flower-like structure. Figure 2e shows a selected area electron diffraction (SAED) pattern recorded on a single sheet. A set of well-defined dots indicates that the NiO films are single crystal in nature. Figure 2f is a higher magnification TEM image showing that a large number of nanowires are closely “woven” together. Figure 2g shows that the nanowires emanating from the nanosheet have a diameter of ~3 nm. Figure 2h shows a TEM image of the inner surface of one nanosheet, in which the nanowires can be observed. The nanowire diameters can be measured based on the intensity profile along a straight line, as shown in the inset of Fig. 2h, demonstrating that the nanowire diameter in the NiO nanosheet is around 3.0 nm. It should be noted, for the products obtained with 1.0 g SDS, the microflower morphology was somehow maintained and observed with thicker nanosheets (Supplementary, Fig. S6).

Other processing factors, such as reaction time and temperature, were also investigated (Supplementary, Fig. S7, S8). A shorter time (1 h) seems to be insufficient to form a well-defined nanosheet structure (Fig. S7a). The microflowers form with a reaction time of 5 hours (Fig. S7b) or 10 hours (Fig. S7c), while some nanosheets peeled off from the hierarchical structures for the reaction time of 20 hours (Fig. S7d). The optimum temperature for forming microflowers was 150 °C. At a temperature of 120 °C, a microsphere structure with nanowires or small nanosheet building blocks was obtained (Fig. S8a), while only nanoribbon structures were formed at 180 °C (Fig. S8b).

### Interconnected Ni-network modified NiO microflowers

**Figure 3:**
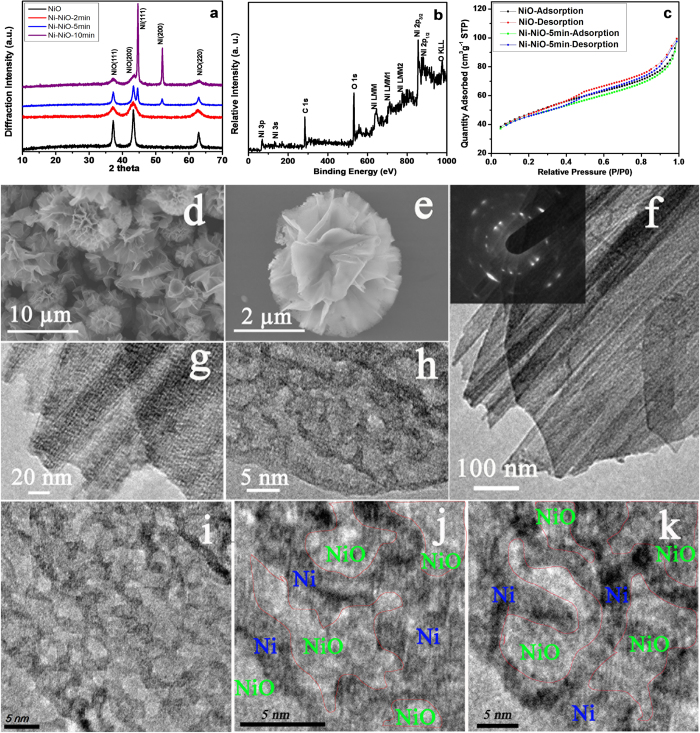
(**a**) XRD patterns of pure NiO microflowers and reduced Ni-NiO products; (**b**)XPS spectrum of the Ni-NiO-5 min sample; (**c**) Nitrogen adsorption/desorption isotherms of pure NiO microflowers and the Ni-NiO-5 min sample; (**d**, **e**) SEM images of the Ni-NiO-5 min sample; (**f**, **g**) TEM and (**h**) HRTEM images of nanosheets in the Ni-NiO-5 min sample; inset of (**f**) is the corresponding SAED pattern; (**h**, **i**) HRTEM images of the Ni-NiO-5 min samples with marked Ni and NiO crystalline region.

While the open hierarchical texture in NiO microflowers facilitates access of ions to the electrode/electrolyte interface (for supercapacitor applications), the poor electrical conductivity may severely retard or even prevent electron transport, leading to a limited use of active material and a significantly lowered specific capacitance. In an attempt to improve electrical conductivity, the as-prepared NiO microflowers were partially reduced to Ni by a controlled heat-treatment under an H_2_/Ar atmosphere (see Methods for details). Figure 3a shows powder X-ray diffraction (XRD) patterns of the as-produced NiO microflowers and the corresponding samples (Ni-NiO) obtained with reduction times of 2, 5, and 10 minutes. All the samples show three peaks at 2θ = 37.2°, 43.2°, and 62.6°, which are indexed as (111), (200), and (220) peaks from the cubic structured NiO (JCPDS No. 47-1049). The samples subjected to H_2_ treatment have peaks at 44.7° and 51.9°, which we index as the (111) and (200) peaks of face-centered-cubic (fcc) Ni. These two Ni peaks increase in intensity relative to the three NiO peaks with increased time of exposure to H_2_(g). As confirmed by SAED pattern, the as-prepared NiO microflower has a single crystal structure. After partial reduction, the single crystal structure was destroyed with the formation of metallic Ni nanoparticles in Ni-NiO-2min, leading to the decrease of NiO XRD peaks in intensity because of the destruction of single crystalline structure of the NiO, and broadening of XRD peaks in width due to the appearance of metallic Ni nanoparticles. With further reaction, more NiO is converted to metallic Ni; consequently the Ni-NiO-5 min shows a remarkable increase in metallic Ni peaks. The Ni-NiO-10 min shows strong metallic Ni peaks and weak NiO peaks, suggesting that most of NiO is converted to metallic Ni.

X-ray photoelectron spectroscopy (XPS) was used to study the chemical states of bonded elements in the Ni-NiO-5 min sample (reduction time of 5 min). The survey XPS spectra indicate C1s (285.1 eV), O1s (531.6 eV), and Ni2p peaks (Fig. 3b). The O 1s and C 1s peaks are attributed to the O in NiO and the substrate (carbon tape), respectively. The Ni2p3/2 curve-fitted data for both Ni-NiO-5 min and pristine NiO samples (Supplementary, Fig. S9a and 9c) indicate the presence of Ni(II) with a main peak at 855.7 eV and a satellite peak at 861.6 eV. A shoulder peak at 852.8 eV is observed for Ni-NiO-5 min, suggesting the presence of metallic nickel. Moreover, the O 1s spectra for both samples were studied (Supplementary, Fig. S9b and 9d). The fitted peak with a binding energy at 530.8 eV is assigned to NiO and the peak at 532.7 eV is assigned to hydrous species. Obviously, the Ni-NiO-5 min, because of the partial reduction of its surface, is much less hydrated than the pristine NiO. According to a controlled TGA experiment, the content of Ni in Ni-NiO-5 min samples is estimated as 18.4 wt.% (Supplementary, Fig. S10). The Ni-NiO-5 min sample shows a similar nitrogen adsorption-desorption curve to the pristine NiO (Fig. 3c); the Ni-NiO-5 min sample has a BET surface area of 151.6 m^2^ g^−1^ and the pristine NiO microflower has a value of 159.6 m^2^ g^−1^. Both samples have an average pore size of around 4.0 nm (Supplementary, Fig. S11). The surfaces and overall structure remain almost unchanged after the hydrogen treatment.

SEM and TEM were performed to investigate the morphology and structure of the samples subjected to H_2_ treatment. The **Ni-NiO-2min** maintained the microflower morphology and showed a polycrystalline structure with lots of metallic Ni nanoparticles decorating the surface (Supplementary, Fig. S12a-c). A typical SEM image of the Ni-NiO-5 min sample (Fig. 3d) shows that the microflower morphology indeed remains essentially the same after partial reduction with H_2_(g). Figure 3e shows the SEM image of a single microflower that has many pores especially at the ridges of the nanosheets; these are due to reduction of the NiO nanosheet surfaces. Figure 3f shows the TEM image of a “petal” in a microflower from the Ni-NiO-5 min sample, and one sees a large number of pores on the nanosheet surface. Two types of polycrystalline rings can be observed based on the SAED analysis (inset of Fig. 3f) and fit for cubic NiO and fcc Ni, consistent with the XRD results. Figure 3g shows a magnified TEM image of the Ni-NiO-5 min sample, in which the ‘woven nanowires’ with a diameter of ~3 nm can be clearly observed. Figures 3h,i show high-resolution TEM image from different nanosheets in the Ni-NiO-5 min microflower. Many shallow pores with irregular shape are present on the sheet surfaces, and these concave pores are surrounded by an interconnected network that was produced after the partial reduction of the nanosheet surface. The magnified HRTEM images were further studied (Figs 3j–k, and Supplementary Fig. S13a-f), revealing that the shallow pores and the network actually belong to two different crystalline structures. One is metallic Ni with a lattice spacing of 0.19 nm, which most commonly appears in the interconnected networks; the other is NiO with a lattice spacing of 0.24 nm that is present on the concave pores of the nanosheets. The **Ni-NiO-10** **min** also basically retained the microflower morphology with a polycrystalline structure and possessed a large amount of pores in the nanosheet surface (Supplementary, Fig. S12d-f), which can be attributed to the fact that most NiO was converted to Ni.

### Supercapacitor performance

**Figure 4:**
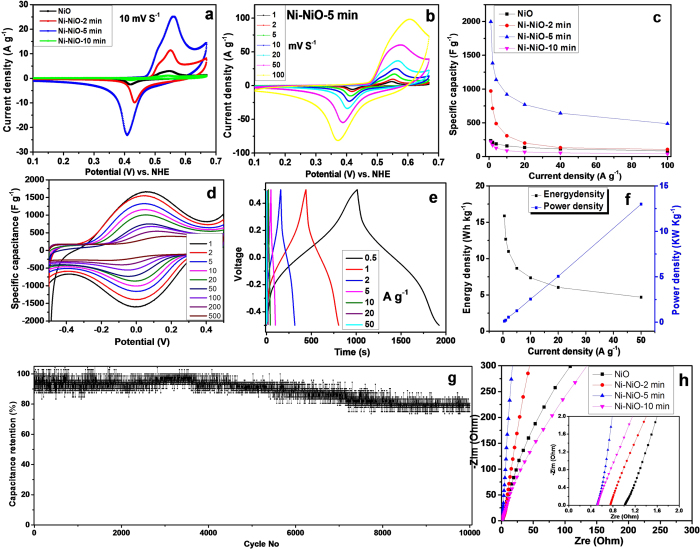
(**a**) CVs of different NiO-based electrodes in 1 M KOH at a scan rate of 10 mV s^−1^ in a three-electrode system; (**b**) CVs of Ni-NiO-5 min electrode in a 3-electrode cell at different scan rates; (**c**) specific capacitance dependence on current density in different NiO-based electrodes of a 3-electrode cell; (**d**) CVs of a two-electrode cell based on Ni-NiO-5 min with different scan rates; (**e**) Galvanostatic charge/discharge curves of a two-electrode cell based on Ni-NiO-5 min at different current densities; (**f**) energy and power densities versus current density for a Ni-NiO-5 min electrode; (**g**) capacitance retention of the Ni-NiO-5 min electrode over cycling at a current density of 5 A g^−1^. (**h**) Nyquist plots for two-electrode supercapacitor cells assembled with different NiO-based materials.

A three-electrode system was used to evaluate the electrochemical properties of the NiO microflower material and the series of Ni-NiO materials. Figure 4a shows cyclic voltammogram (CV) curves of different electrodes in a 1.0 M KOH solution at a scan rate of 10 mV s^−1^. A pair of peaks is observed in the range of 0.35–0.55 V for all curves arising from the following redox reaction of NiO:


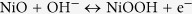


The Ni-NiO-2 min sample had a three times higher peak current density compared with the pristine NiO, and the Ni-NiO-5 min sample had an order of magnitude higher peak current density than the pristine NiO. Because most of the NiO was converted into Ni, the Ni-NiO-10 min sample showed a much smaller peak current density than the pristine NiO. The capacitance was calculated by integrating the area of the CV curve. The pristine NiO microflower material had a specific capacitance of 100.5 F g^−1^, a much smaller value than the theoretical value of NiO (2,584 F g^−1^). The Ni-NiO-2 min sample gravimetric capacitance was 388.9 F g^−1^, the Ni-NiO-5 min sample 964.9 F g^−1^, and the Ni-NiO-10 min sample 45.6 F g^−1^.

Figure 4b shows a series of CV curves at various scan rates for the Ni-NiO-5 min sample. At a scan rate of 1 mV s^−1^, the specific capacitance was 1,579 F g^−1^, while that of the pristine NiO was 149.1 F g^−1^ and that of Ni-NiO-2 min was 754.4 F g^−1^. The Ni-NiO-5 min sample had the highest capacitance among the different samples at the same scan rate (Supplementary, Fig. S14), which delivered a capacitance of 515.4 F g^−1^ at a scan rate of up to 100 mV s^−1^, while the pristine NiO and the Ni-NiO-2 min samples had lower values of 55.3 F g^−1^ and 225 1 F g^−1^, respectively. The specific capacitance was also calculated based on the galvanostatic charge/discharge curves within a potential window of 0.1 ~ 0.67 V (*vs.* NHE) (Supplementary, Fig. S15); the Ni-NiO-5 min sample had a significantly improved capacitance compared with the other three NiO-based samples at the same current density, as reflected in Fig. 4c.

CV and galvanostatic charge-discharge tests were further performed with a symmetrical two-electrode cell, which could provide reliable measurements for evaluating supercapacitors’ performance^44^. It should be noted that for a two-electrode cell, we did not observe the pair of charge-discharge plateau in the range of 0 ~ 05 V, which should be present in the NiO system except for a charge plateau in the first charge process, suggesting that only oxidation reactions occur in the first scan. Therefore, the electrochemical tests were conducted within a −0.5 to 0.5 V voltage window. Figure 4d shows rate-dependent CV curves of the supercapacitor with Ni-NiO-5 min at various scan rates from 1.0 to 500 mV/s. A pair of redox peaks between −0.1 and 0.1 V can be seen, which correspond to redox conversion reactions between NiO and NiOOH. The specific capacitance for the Ni-NiO-5 min is substantially larger than that based on pristine NiO at the same scan rates (Supplementary, Fig. S16a). The improved electrochemical performance was confirmed by galvanostatic charge-discharge measurements performed at different current densities in a two-electrode cell. Figure 4e shows the galvanostatic charge/discharge curves of a Ni-NiO-5 min symmetrical supercapacitor at different current densities. Two plateaus corresponding to redox reactions of NiO can be identified in the curves, indicating that the electrode material has a good pseudocapacitive characteristic and excellent electrochemical reversibility. The two-electrode cell based on Ni-NiO-5 min delivered specific capacitances of 1,828, 1,460, 1,266, 999, 848, 696, and 540 F g^−1^ at current densities of 0.5, 1.0, 2.0, 5.0, 10.0, 20.0, and 50.0 A g^−1^, respectively. Further analyses show that the Ni-NiO-5 min has a significantly higher capacitance and better rate capability than a two-electrode supercapacitor based on pristine NiO (Supplementary, Fig. S16b, c).

Figure 4f shows the relation between the current density and the energy density as well as the power density. The Ni-NiO-5min sample had the following characteristics: (i) An energy density as high as 15.9 Wh kg^−1^ at 0.5 A g^−1^, corresponding to 1,828 F g^−1^ and a power density of 0.12 kW kg^−1^; (ii) For 20 A g^−1^, an energy density of 6.0 Wh kg^−1^ and a power density of 5.1 kW kg^−1^; (iii) At 50 A g^−1^, an energy density of 3.1 Wh kg^−1^ and a power density of 13.2 kW kg^−1^. The Ni-NiO-5 min sample, with a bulk-density of 0.74 g cm^−3^, had a volumetric specific capacitance of 1,352.7 F cm^−3^ at 0.5 A g^−1^. The electrochemical stability was evaluated using the galvanostatic charge-discharge method on the Ni-NiO-5 min-based 2-electrode symmetrical supercapacitor at a current density of 5.0 A g^−1^ (Fig. 4g). It retained more than 80% of its initial capacitance after 10,000 cycles, demonstrating that the Ni-NiO-5 min sample has reasonable electrochemical stability and cycle reversibility.

In order to further confirm the influence of the reduction treatment on the supercapacitor performance, electrochemical impedance spectroscopy (EIS) was performed on the series of NiO-based supercapacitors. The results are presented in terms of Nyquist plots in Fig. 4h. The equivalent series resistance (ESR), which is a measure of conductivity related to the resistance of an electrode material, can be calculated from the intercept of the corresponding Nyquist plots on the Z real-axis. All supercapacitors exhibit an ESR value lower than 1.5 Ω, suggesting that the nickel foam provides an excellent current collector for active materials. The order of the intercept on Z real-axis for the four supercapacitors is: Ni-NiO-10 min (0.53 Ω) < Ni-NiO-5 min (0.56 Ω) < Ni-NiO-2 min (0.78 Ω) < pristine NiO (1.03 Ω). The ESR value for these samples are consistent with the reduction level, indicating that the H_2_ treatment did contribute to reduce the ESR of NiO microflowers. In addition, in the low frequency region, the **Ni-NiO-5** **min** sample exhibits a near-vertical Nyquist line, while the pristine NiO and Ni-NiO-10 min shows more inclined Nyquist lines. The more vertical curve in a cell suggests easier diffusion of ions and more ideal capacitive behavior.

## Discussion

**Figure 5:**
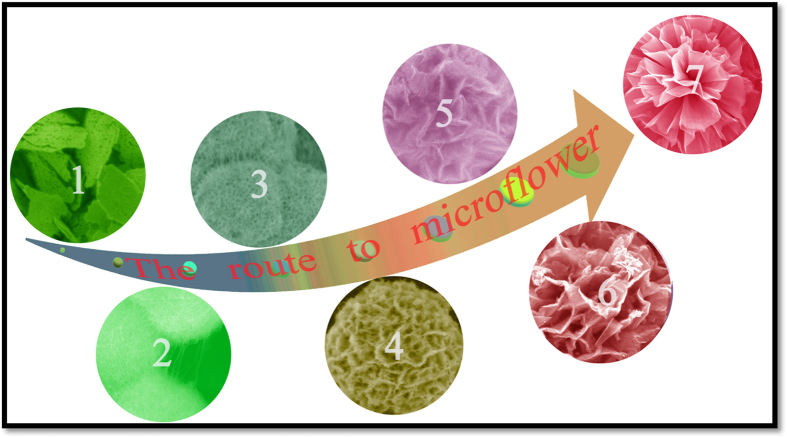
SEM images of the series of NiO products obtained under different experimental conditions that demonstrate the “weaving” evolution process. NiO samples obtained at: (**1**) a ratio NiCl_2_/Urea of 5:1, without SDS; (**2**) a ratio NiCl_2_/Urea of 3:1, without SDS; (**3**) a ratio NiCl_2_/Urea of 1:1, without SDS; (**4**) a ratio NiCl_2_/Urea of 1:1, 0.1 g SDS; (**5**, **6**) a ratio NiCl_2_/Urea of 1:1, 0.3 g SDS; (**7**) a ratio NiCl_2_/Urea of 1:1, 0.6 g SDS. The synthesis was carried out at 150 °C with a hydrothermal reaction time of 5 hours.

Although hierarchical 3D NiO microstructures were reported previously^37^,^38^,^39^,^40^,^41^,^42^,^43^, an understanding of the formation of such unique microstructures is still lacking. Both SEM and TEM analysis prove that the NiO microflowers are actually composed of highly ordered nanowires. SEM images of typical products obtained under different conditions together demonstrate a clear and intuitive vision of the evolution process of the microflowers, as shown in Fig. 5. The morphology changes from a microsphere built of nanowires, to a porous nanowire microsphere framework, and finally to microflowers constructed from nanosheets. These results provide strong evidence that the nanosheet building block in a Ni-based microflower has evolved from a nanowire “weaving” process.

Apparently, urea and SDS are two key ingredients in the formation of microflowers constructed from NiO nanosheets. The hydrolysis of urea under hydrothermal conditions generates ions (e.g., OH^−^, CO_3_^2−^) that are indispensable for precipitating Ni^2+^. In addition, the concentration of urea can significantly affect the morphology of the Ni-based products, indicating that urea also functions as a structure-directing agent to confine the growth of microspheres from nanoplates to nanowires. On the other hand, SDS acts as a “binder” for “weaving” these nanowires into large single-crystalline sheets that finally build a microflower. It is well-known that urea has two −NH_2_ groups joined by a carbonyl (C = O) functional group that tends to form a dense and energetically favorable hydrogen-bond network, which is probably critical to the formation of a Ni-based microsphere constructed from nanowires. Additionally, by virtue of its tendency to form a porous framework, urea also has the ability to trap anionic surfactants of SDS compounds and hold them in the channels formed by interpenetrating helices composed of hydrogen-bonded urea molecules^45^,^46^. The hydrophilic group of SDS then binds with Ni^2+^ or Ni-based complexes through static interactions and further induces the ultrahigh density parallel assembly of Ni-based nanowires^13^,^47^ to subsequently “weave” them into nanosheets within the porous microsphere frameworks and finally evolve into a microflower structure (Supplementary, Fig. S17). It should be noted that the reduction reaction of highly “woven” NiO nanowires results in the formation of a conductive network of metallic Ni, which is most likely due to rearrangement of Ni nanoparticles on the surfaces of the NiO.

It is known that the charge storage reactions in NiO-based supercapacitors are highly dependent on the electrode surface layer. Microflowers constructed from thin-layer nanosheets offer an ideal nanostructure to maximize the exposed active surface area at electrode/electrolyte interfaces, which makes it possible to make full use of the NiO. However, a large fraction of “dead surface” inevitably exists due to the poor electrical conductivity of NiO^25^,^26^,^27^,^28^,^29^, as demonstrated by the low measured capacitance in pristine NiO microflowers. We also confirmed that partial reduction of other metal oxides contributed to an improved utilization efficiency of active materials (Supplementary, Figs S18 and S19). In this work, partial reduction of NiO (Ni-NiO-5 min) led to the generation of an interconnected Ni network on its surface, which is completely different from the Ni-NiO nanocomposites^27^,^28^,^29^,^30^ reported previously that are normally core-shell structures and do not have maximum access to electrolytes (by the active NiO material). First of all, the interconnected Ni network provides a network channel for electron transfer on the nanosheet surface and thus significantly improves the electrical conductivity. In addition, such an open hierarchical microflower enables both sides of every nanosheet to easily access ions/electrolyte for the redox reaction between NiO and NiOOH. And as a result, the Ni-NiO-5 min electrodes manifested a higher utilization efficiency, as evidenced by negligible decreases in specific capacitance values with increasing mass loadings (Supplementary, Fig. S20). Moreover, the microflower structure can be beneficial to prevent agglomeration and to maintain the structural integrity because microflowers can accommodate the mechanical stress resulting from the volume change upon cycling. Therefore, the near-optimum Ni-NiO-5 min sample had a capacitance of around 1,828 F g^−1^ at a current density of 0.5 A g^−1^, with respectable power density and energy density. Notably, the capacitance of Ni-NiO-5 min is calculated as 2,240 F g^−1^ based on the mass of NiO, which almost approaches its theoretical capacitance (2,584 F g^−1^). In fact, the as-developed Ni-NiO-5 min outperforms nearly all NiO nanostructures reported previously in terms of various supercapacitor performance indices (Supplementary, Table S1). It should be pointed out that both the bare carbon cloth and bare nickel foam current collectors delivered a capacitance less than 1.0 F g^−1^ (Supplementary, Fig. S21 and S22), confirming that the high capacitance originates from the Ni-NiO-5 min sample itself instead of the Ni-foam current collector. Furthermore, based on results of the two-electrode planar symmetrical supercapacitor (coin cell CR2032), the Ni-NiO-5 min can deliver a capacity of around 70 mAh g^−1^ at a current density of 0.5 A g^−1^ and 19 mAh g^−1^ even at a high current density of 50 mA g^−1^ (Supplementary, Fig. S23), and holds an energy density approaching those of conventional batteries without sacrificing the high power that a supercapacitor typically offers.

To conclude, based on many experiments a ‘weaving’ mechanism is proposed for the first time to illustrate the formation of the hierarchical NiO structures with multiple characteristic dimensions: the 3D microflower was constructed from 2D nanosheets derived from a weaving process of 1D nanowires. An interconnected conductive Ni network on the surface of the nanosheet ‘building blocks’ of the microflower was produced through partial reduction of the NiO, rendering the material more electrically conductive and significantly improving the supercapacitor performance. Our approach may pave the way for understanding the formation mechanism of such ‘microflower’ nanostructures and further advancing other metal oxide-based electrode materials for supercapacitors with outstanding performance.

## Methods

### Materials and Synthesis

All chemicals were analytical grade and were purchased from Sigma-Aldrich and used without further treatment. The NiO microflower sample was prepared using a hydrothermal method followed by calcination. In a typical experiment, 0.6 g of sodium dodecyl sulfate (SDS) was dissolved in 60 ml water at room temperature to form a homogeneous and clear solution. 5.0 mmol urea was then added to the solution under drastic stirring. Afterwards, 5.0 mmol nickel chloride (NiCl_2_) was slowly added to this solution with vigorous agitation. The solution was then transferred to a 100 ml Teflon-lined stainless steel autoclave and heated at 150 °C for 5 h. The obtained green precipitates were filtered and washed six times with distilled water and absolute alcohol to remove all soluble materials. After drying the products were calcined at 500 °C for two hours. The NiO products were initially heated to 350 °C at a rate of 5 °C/min in argon and maintained at 350 °C with a flow of H_2_/Ar (1:9) gas for 5 min. The partially reduced product was automatically cooled under an Ar atmosphere. Major chemical reactions involved in the synthesis include:


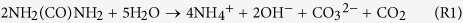



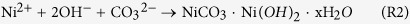



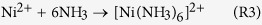



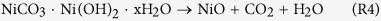



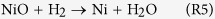


### Characterization

The structures and morphologies of the as-prepared samples were obtained using a Hitachi H-800 TEM and a LEO 1530 SEM. XRD was performed on a Bruker D8-Advance X-ray powder diffractometer. Specific surface areas were measured at 77 K by BET nitrogen adsorption-desorption (Shimadzu, Micromeritics ASAP 2010 Instrument). To investigate the decomposition process of NiCO_3_∙Ni(OH)_2_ ∙ xH_2_O, the as-obtained hydrothermal samples were analyzed by thermogravimetric analysis (TGA, Mettler Toledo TGA-SDTA851 analyzer) from 25 to 500 °C under an air atmosphere with a heating rate of 5 °C/min. To analyze the content of metallic Ni in Ni-NiO, TGA analysis was carried out on the Ni-NiO-5 min samples by first heating them to 300 °C with a heating rate of 5 °C/min and maintaining them at 300 °C for 30 minutes under Ar protection, which can eliminate the effect of adsorbed water; the samples were then heated to 450 °C in air to investigate the oxidation reaction of metallic Ni. X-ray photoelectron spectroscopy (XPS) was carried out on VG ESCA 2000 with Mg K*α* as the X-ray source and the C1s peak at 284.6 eV as an internal standard.

### Electrochemical Measurements

Electrochemical measurements were conducted using a three-electrode system with a saturated calomel electrode (SCE) and Pt gauze as the reference and the counter electrodes, respectively. Working electrodes were prepared by mixing NiO samples, carbon black (5 wt.%), and poly-vinylidene fluoride (PVDF, 5 wt.%) in N-Methylpyrrolidone (NMP) to form a slurry, which was then pasted onto carbon cloth and dried at a temperature of 80 °C in a vacuum oven. Cyclic voltammetry was conducted using a CHI 760 electrochemical station in 1.0 M KOH aqueous electrolytes with a voltage range of 0.1 ~ 0.67 V at various scan rates. Electrochemical impedance spectroscopy (EIS) was performed by applying an AC voltage with an amplitude of 5 mV within the frequency range from 100 kHz to 50 mHz on the series of NiO-based two-electrode supercapacitors. Galvanostatic charge-discharge tests were also conducted in a CHI 760 electrochemical station using the same system as that used for the above CV testing. The specific capacitance (C_m_, F g^−1^) was calculated based on CV (Eq. 1) and galvanostatic charge-discharge tests (Eq. 2):


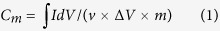



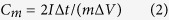


where I is the current (A), V is the voltage, v is the scan rate (V s^−1^), and m is the mass (g) of the active material in the electrode. ΔV is the potential range for the electrochemical process and Δt is the discharge time.

A stainless-steel coin cell (CR2032) supercapacitor with two symmetrical electrodes was assembled in air. To prepare the working electrode, a paste mixture consisting of active materials (Ni-NiO, 90%), acetylene black (5%), and PVDF (5%) was compressed onto a nickel foam current collector (Φ = 0.5 inches). After drying, the pellets with an active material weight of around 0.8 mg were assembled into a symmetrical supercapacitor, in which 1.0 M KOH solution was used as the electrolyte and a hydrophilic Millipore PVDF membrane was used as the separator. Cyclic voltammetry and galvanostatic charge-discharge tests were carried out using CHI 760 electrochemical station and a Land battery tester system. The gravimetric capacitance (F g^−1^) based on the CV curve was calculated as:


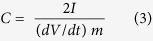


where *I* is the current recorded (unit: A), *dV*/*dt* is the scan rate mV s^−1^, and m is the average mass (g) of the active material in each electrode.

With the galvanostatic charge/discharge plots based on two-electrode cells, the specific capacitance (*C*_m_, unit: F g^−1^) of a single electrode, the energy density (*D*_*e*_, unit: Wh kg^−1^), and the power density (*D*_*p*_, unit: kW kg^−1^) of the supercapacitor were calculated based on Eqs 4, 5, 6:


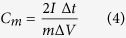



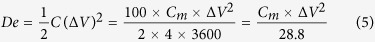



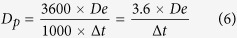


where *I* is the constant discharge current (unit: A), ∆*t* is *t*he discharge time (unit: s), and ∆*V* is the voltage window (0.5 V), and m is the average total mass (g) of the active material in both electrodes. The density of our NiO-based microflower products is ∼0.74 g cm^−3^, and the volumetric capacitance was calculated as:


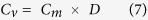


where D is the bulk density calculated as the average of the mass of many particles of the material divided by the total volume they occupy.

## Additional Information

**How to cite this article**: Ci, S. *et al.* NiO-Microflower Formed by Nanowire-weaving Nanosheets with Interconnected Ni-network Decoration as Supercapacitor Electrode. *Sci. Rep.* **5**, 11919; doi: 10.1038/srep11919 (2015).

## Supplementary Material

Supplementary Information

## Figures and Tables

**Figure 1:**
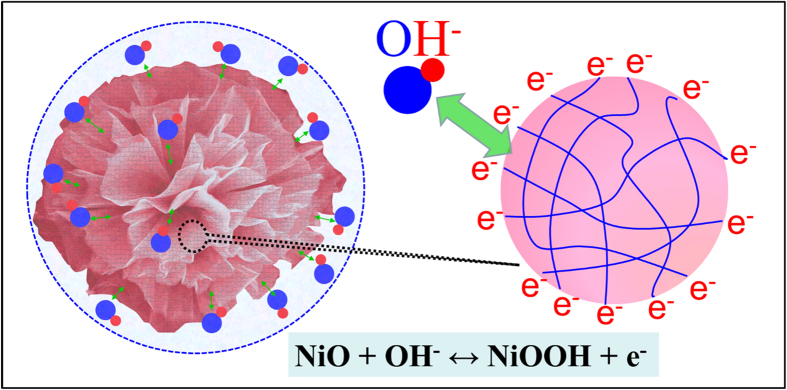
Schematic of the as-designed Ni-NiO microflower used for a supercapacitor.
