# Supplementary material for: NiO-Microflower Formed by Nanowire-weaving Nanosheets with Interconnected Ni-network Decoration as Supercapacitor Electrode
Source: Sci Rep. 2015 Jul 13;5:11919. doi: 10.1038/srep11919 (PMC5387177; doi:10.1038/srep11919)
Supplement: Supplementary Information [file srep11919-s1.pdf]

Supporting Information for：

NiO-Microflower Formed by Nanowire-weaving Nanosheets with Interconnected Ni-network Decoration as Supercapacitor Electrode

Suqin Ci, Zhenhai Wen,* Yuanyuan Qian, Shun Mao, Shumao Cui, and Junhong Chen,*

Department of Mechanical Engineering, University of Wisconsin-Milwaukee, Milwaukee, Wisconsin 53211, United States

Corresponding Author* E-mail: wenzhenhai@yahoo.com, jhchen@uwm.edu.

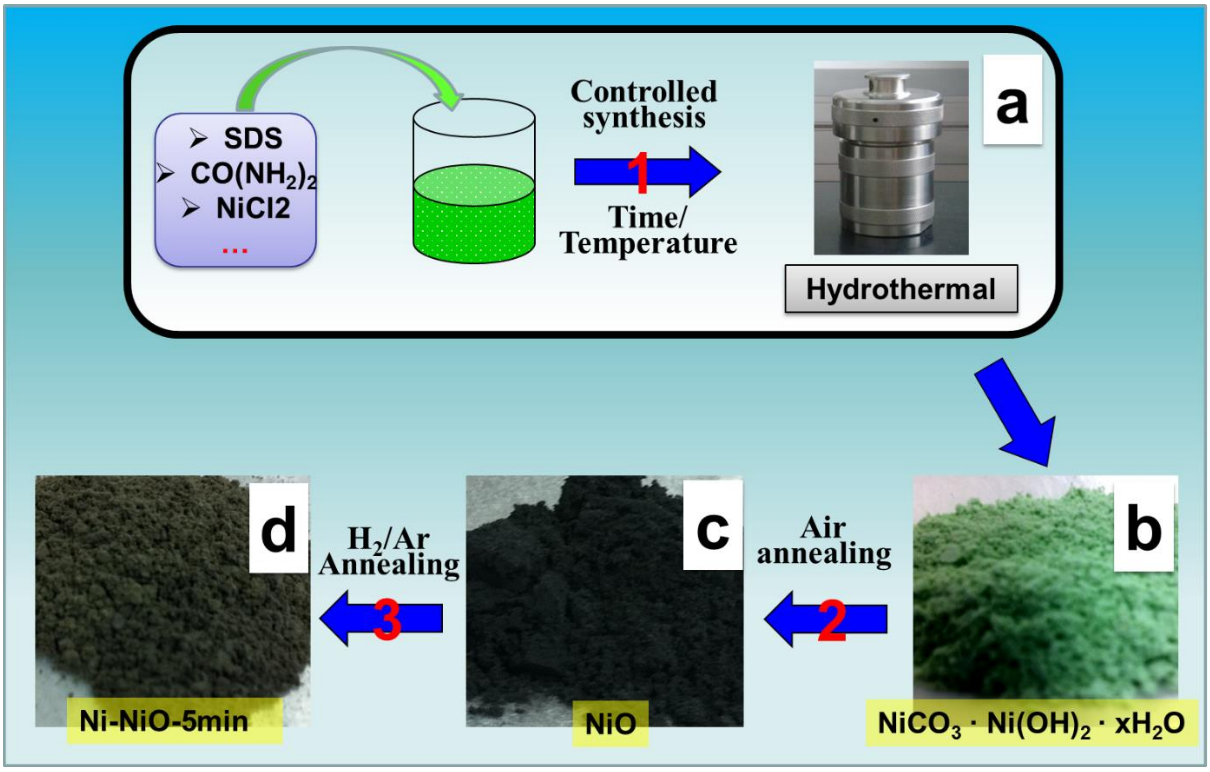

Fig. S1. Schematic of the synthesis of Ni-NiO nanocomposites: (1) hydrothermal synthesis; (2) annealing of the hydrothermal products; (3) partial reduction of NiO under an H_2/Ar atmosphere.

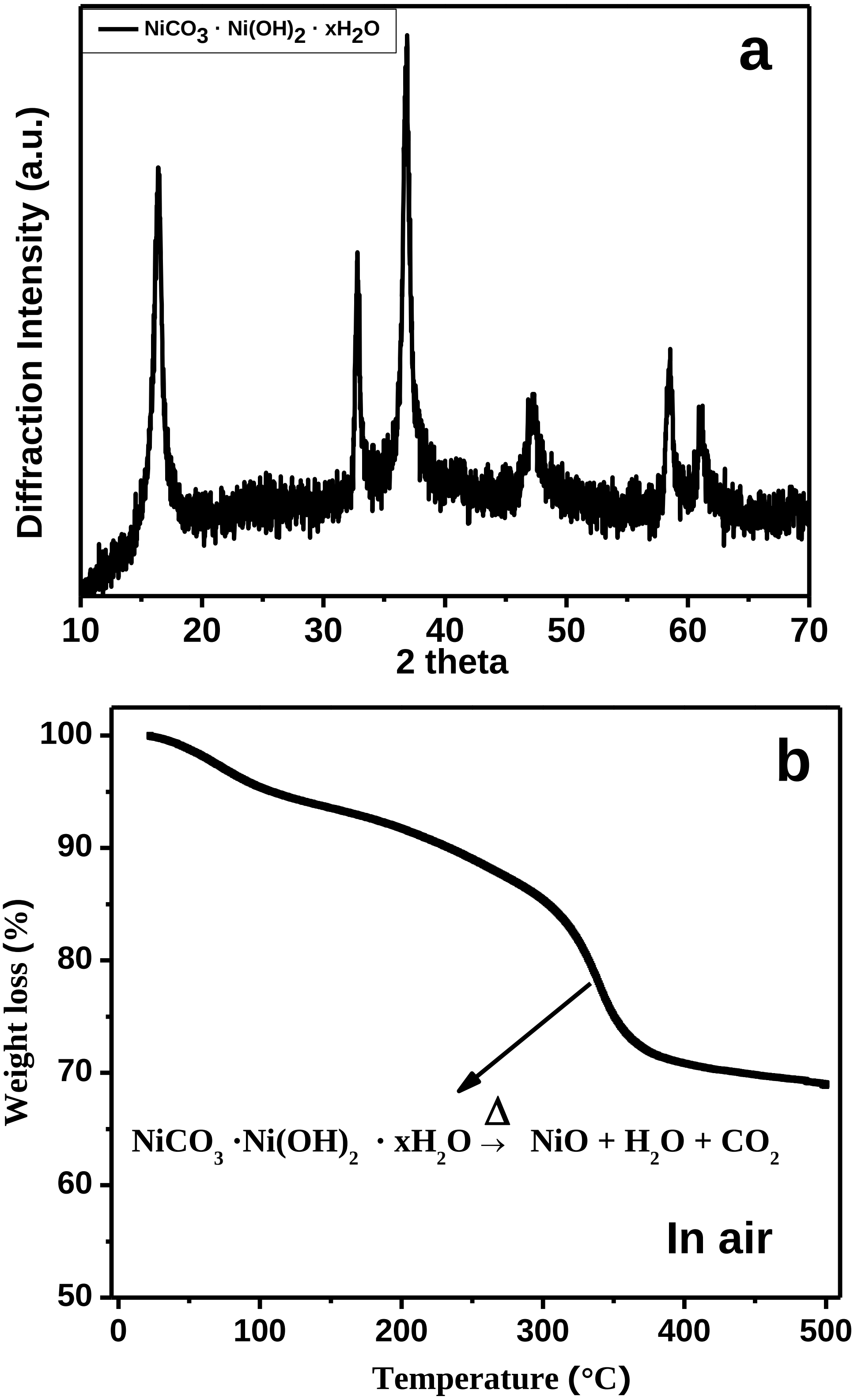

Fig. S2. (a) XRD patterns of the as-prepared hydrothermal product, i.e., $NiCO_3$ $Ni(OH)_2$ xH_2O; (b) Thermal gravimetric analysis (TGA) curve of the as-prepared $NiCO_3$ $Ni(OH)_2$ xH_2O.

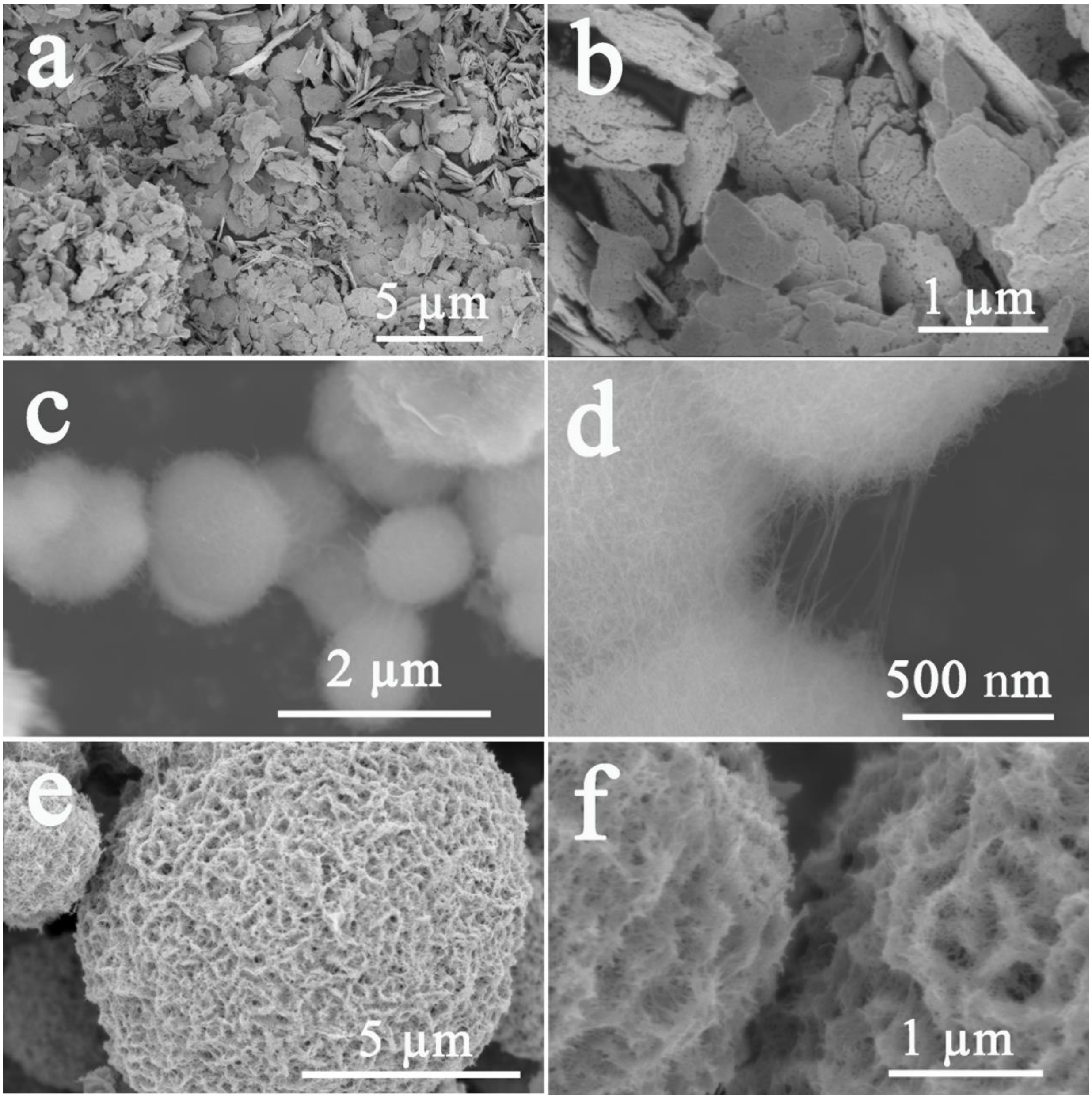

Fig. S3. SEM images of the NiO products obtained with a ratio of $NiCl_2$/Urea: (a, b) 5:1; (c, d) 3:1; (e, f) 1:1; the amount of $NiCl_2$ was fixed at 5 mmol and the reaction temperature was 150 ℃.

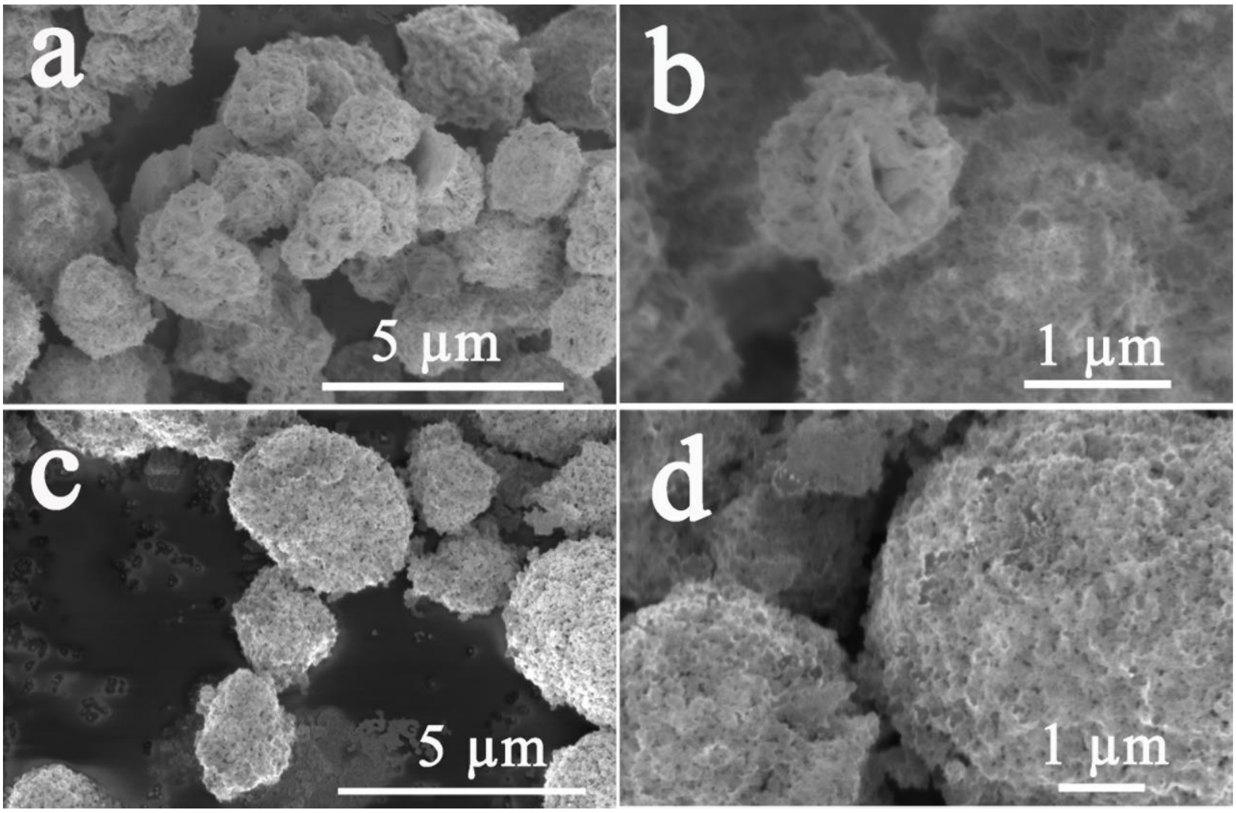

Fig. S4. SEM images of the NiO products obtained with a ratio of $NiCl_2$/Urea: (a, b) 1:2, (c, d) 1:10; the amount of $NiCl_2$ was fixed at 5 mmol and the reaction temperature was 150 ℃.

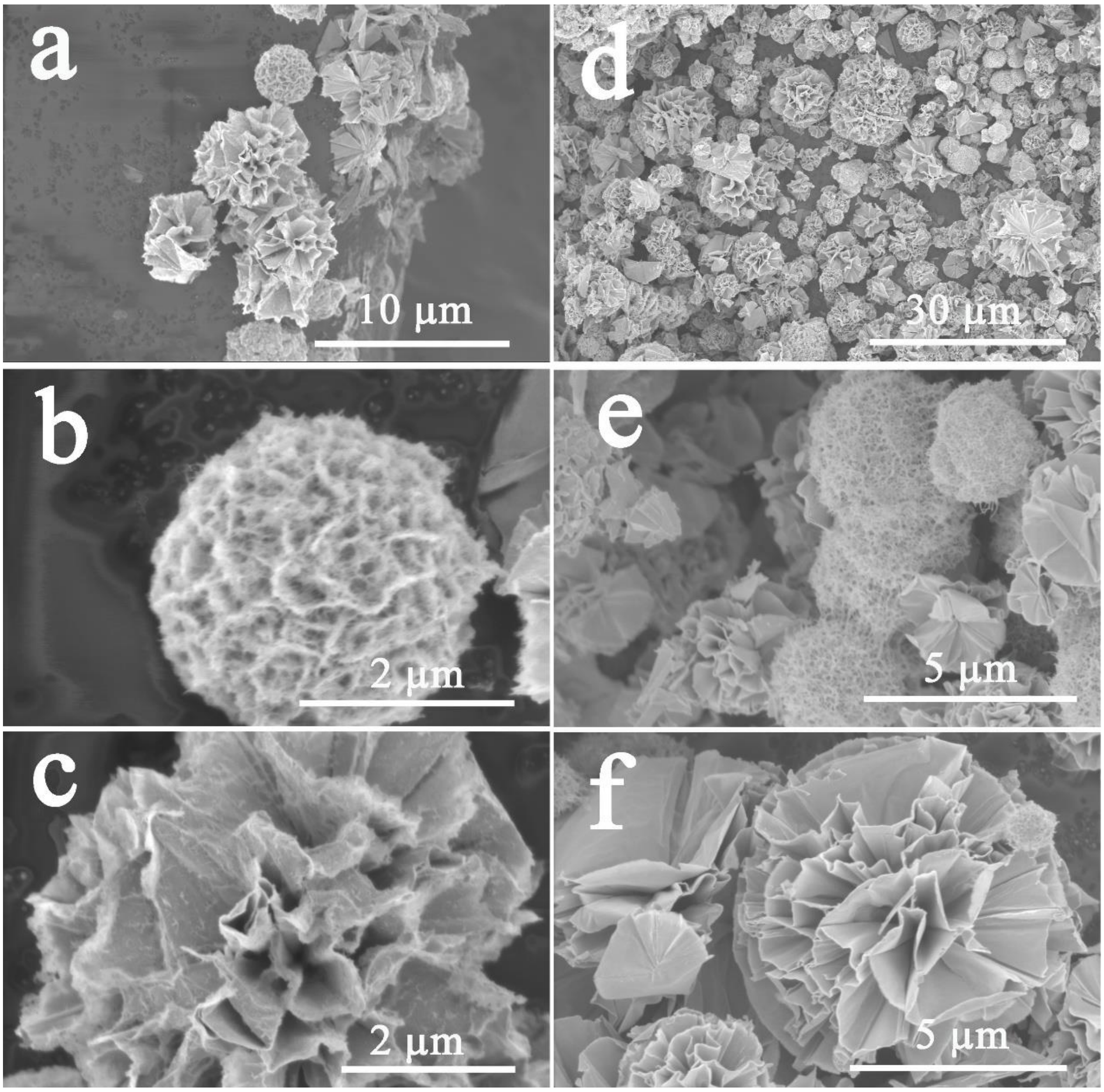

Fig. S5. SEM images of the NiO products synthesized by adding 0.1 g SDS (a-c): (a) the general morphology, (b) the magnified image of porous microspheres constructed from nanowires, and (c) the magnified image of 'microflowers' constructed from nanosheets; SEM images of the NiO products synthesized by adding 0.3 g SDS (d-f): (d) the general morphology, (e) the magnified image of porous microspheres constructed from nanowires, and (f) the magnified image of 'microflowers' constructed from nanosheets; the amount of $NiCl_2$ and urea was fixed at 5 mmol and the reaction temperature was 150 ℃.

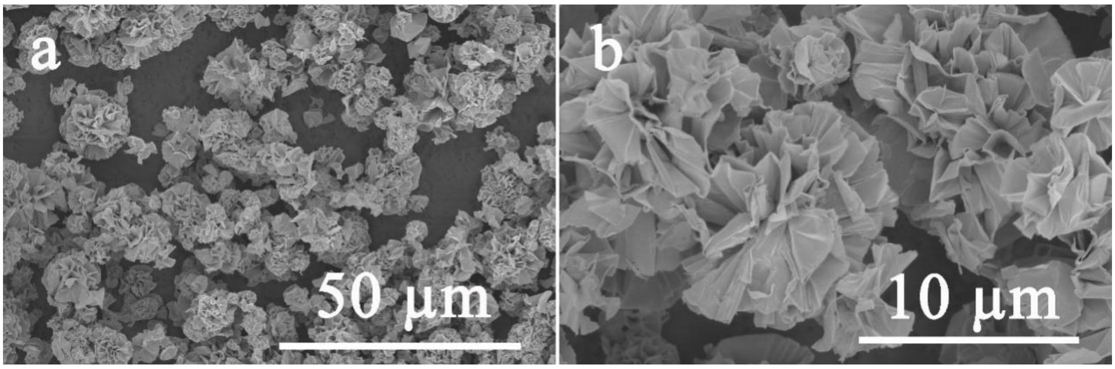

Fig. S6. SEM images of the NiO products obtained when adding 1.0 g SDS; the amount of $NiCl_2$ and urea was fixed at 5 mmol and the reaction temperature was 150 ℃.

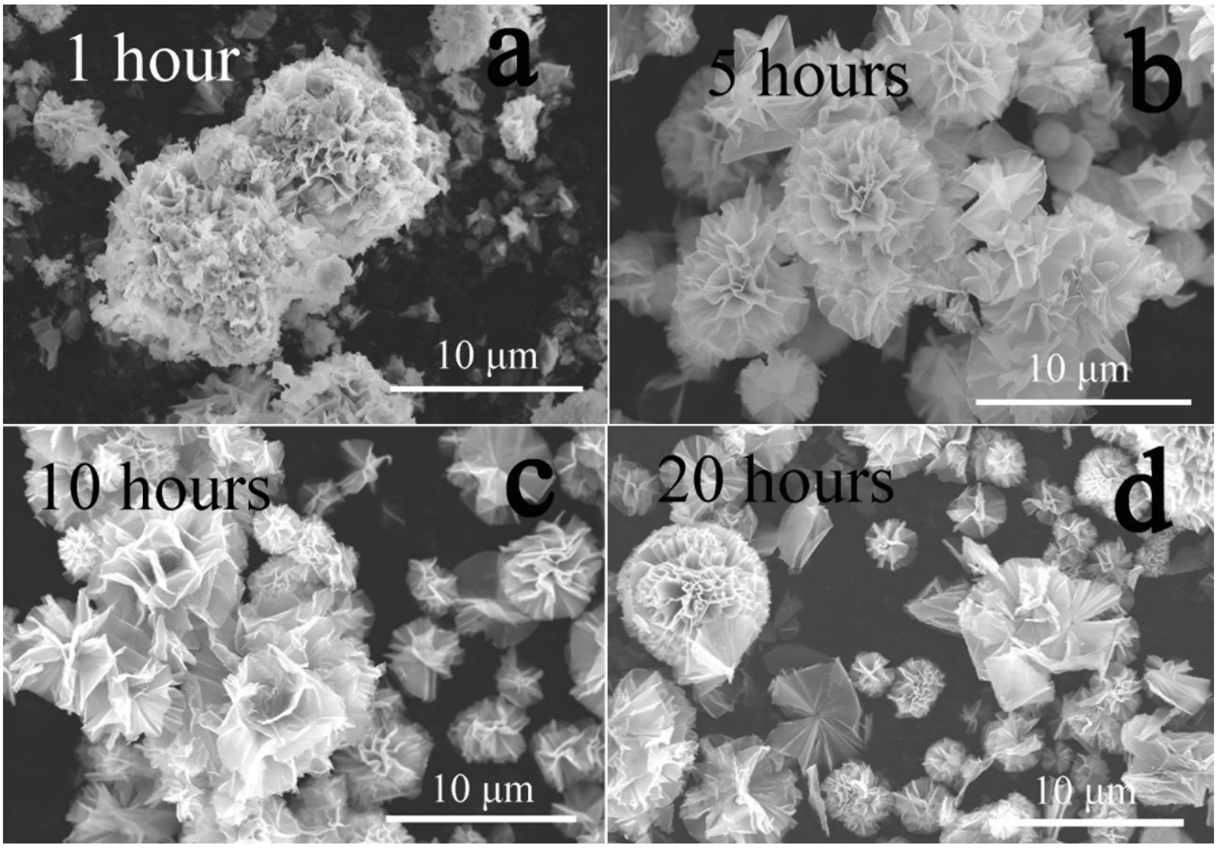

Fig. S7. SEM images of the NiO products obtained with a hydrothermal reaction time of: (a) 1 hour; (b) 5 hours; (c) 10 hours; and (d) 20 hours. (Note: 0.6 g SDS; the amount of $NiCl_2$ and urea was fixed at 5 mmol and the reaction temperature was 150 ℃)

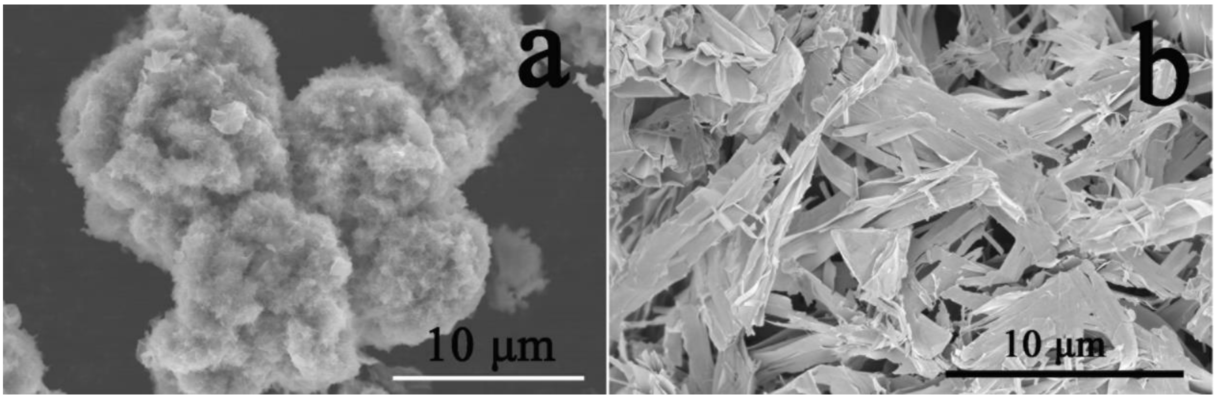

Fig. S8. SEM images of the NiO products obtained with a hydrothermal reaction temperature of: (a) 120 ℃; (b) 180 ℃. (Note: 0.6 g SDS; the amount of $NiCl_2$ and urea was fixed at 5 mmol)

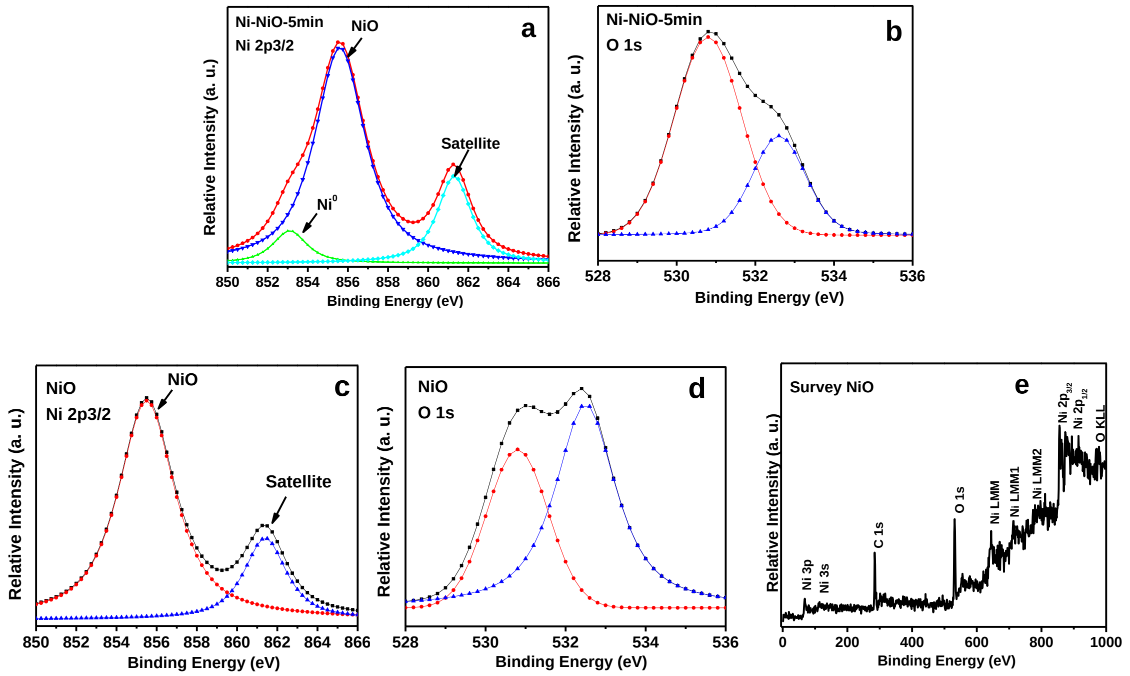

Fig. S9. High resolution XPS spectra of the Ni 2p3/2 (a) and O 1s (b) in Ni-NiO-5min after Shirley background removal; High resolution XPS spectra of the Ni 2p3/2 (c) and O 1s (d) in the pristine NiO after Shirley background removal, and survey XPS spectrum of the pristine NiO. The fitted peak with a binding energy at 531.0 eV is assigned to NiO and the peak at 532.6 eV is assigned to hydrous species.5

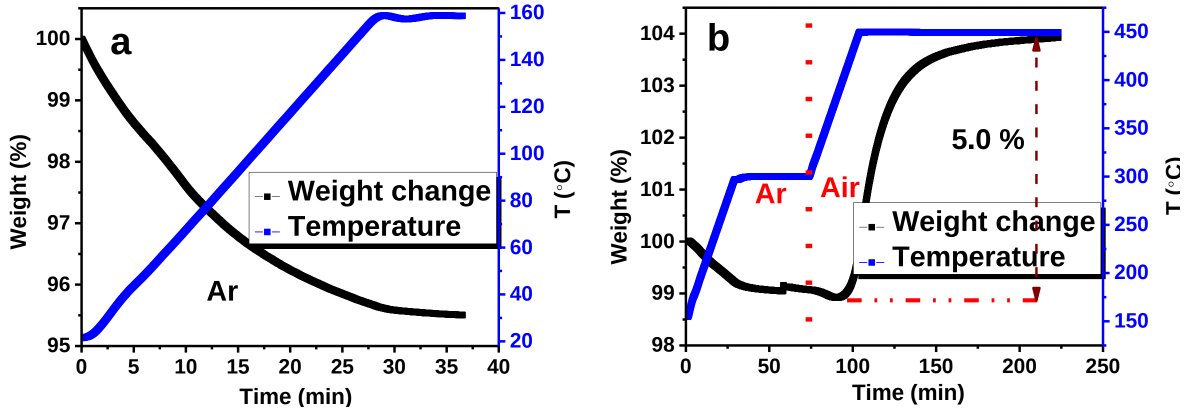

Fig. S10. TGA curve of the as-prepared Ni-NiO-5min upon heating to 160 °C under Ar atmosphere with a heating rate of 5 °C/min (a), and subsequent heating to 300 °C with a heating rate of 5 °C/min and maintaining at 300 ℃ for 30 minutes under Ar protection to eliminate the effect of adsorbed water; the sample was then heated to 450 °C in air to investigate the oxidation reaction of metallic Ni (b).

The Ni content in Ni-NiO-5min was calculated based on the reaction below:

$$Ni + 1/2\ O_2 = NiO$$

$$59 \qquad\qquad 75$$

$$x\ (g) \qquad\qquad 75x/59\ (g)$$

The mass increase is 16x/59, in which 59 and 75 are molecular weights of Ni and NiO, respectively.

Assuming that there is x (g) metallic Ni in 1 g Ni-NiO-5min sample, which would convert to 75x/59 (g) NiO after fully oxidizing to NiO. This means the total weight should increase by 16x/59; i.e., the total weight increases from 1 to 1+16x/59. The TGA test indicated the total weight increases by 5.0% after heating to 450 °C. Therefore (75x/59-x)/1 = 16x/59 = 5.0%, and x is calculated as 0.184, suggesting that there is 18.4 wt.% Ni in the Ni-NiO-5min samples.

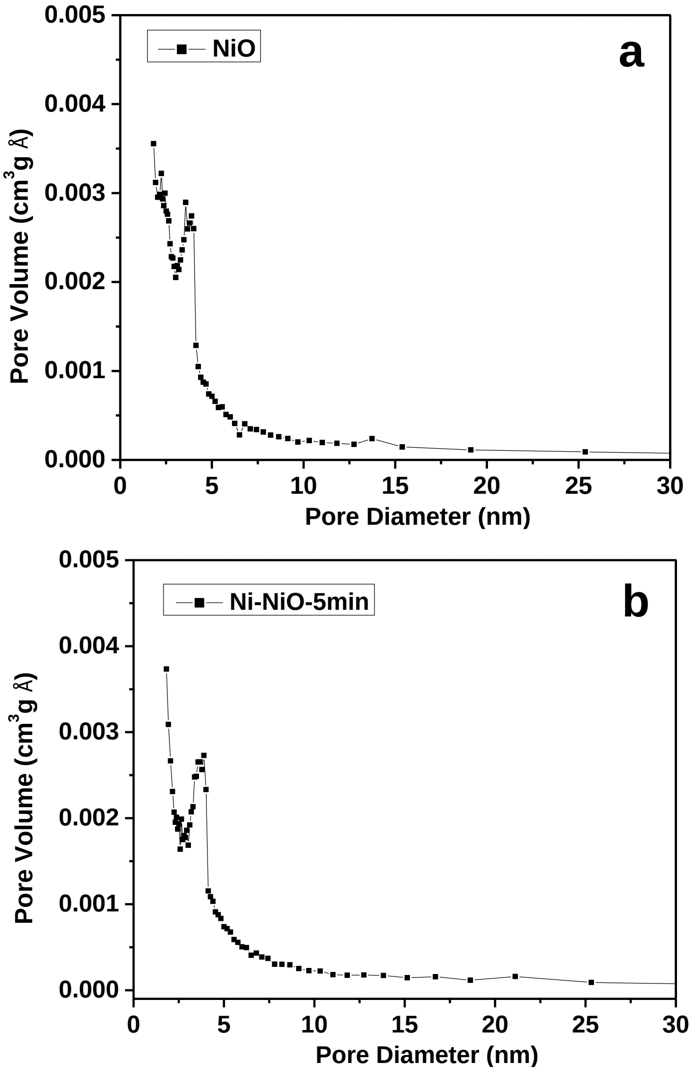

Fig. S11. The pore size distribution of the NiO and the Ni-NiO-5min samples.

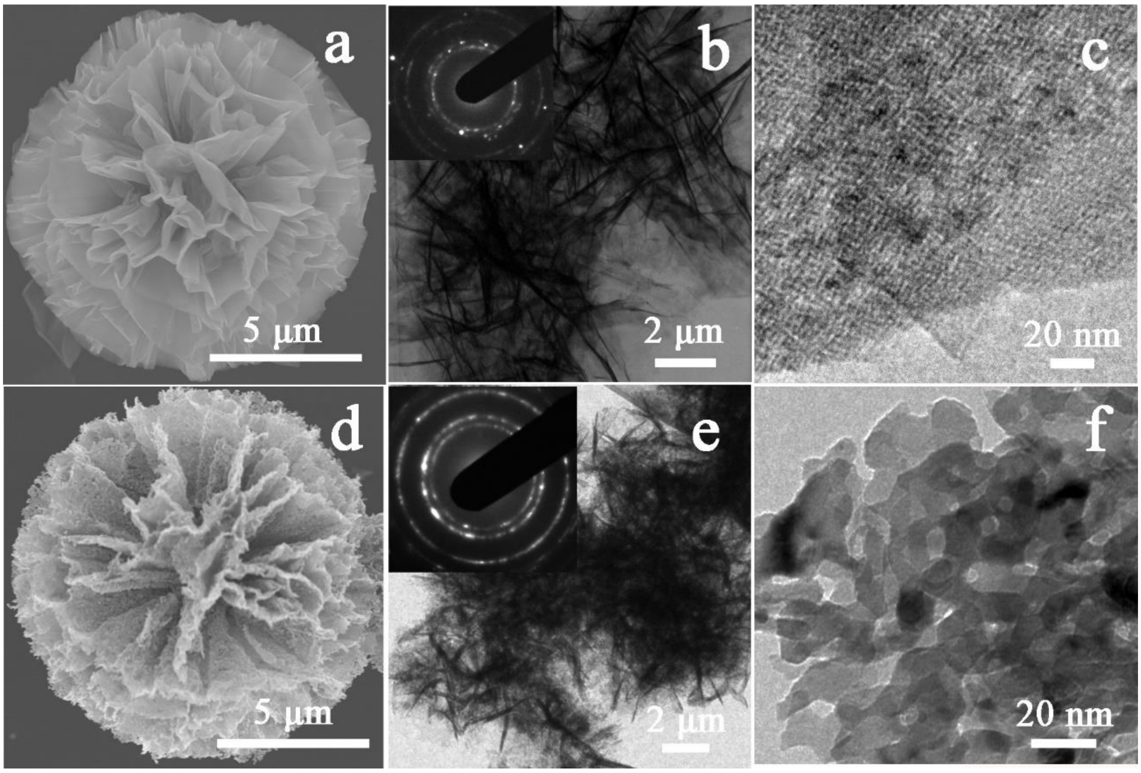

Fig. S12. SEM and TEM images of (a-c) Ni-NiO-2min and (d-f) Ni-NiO-10min.

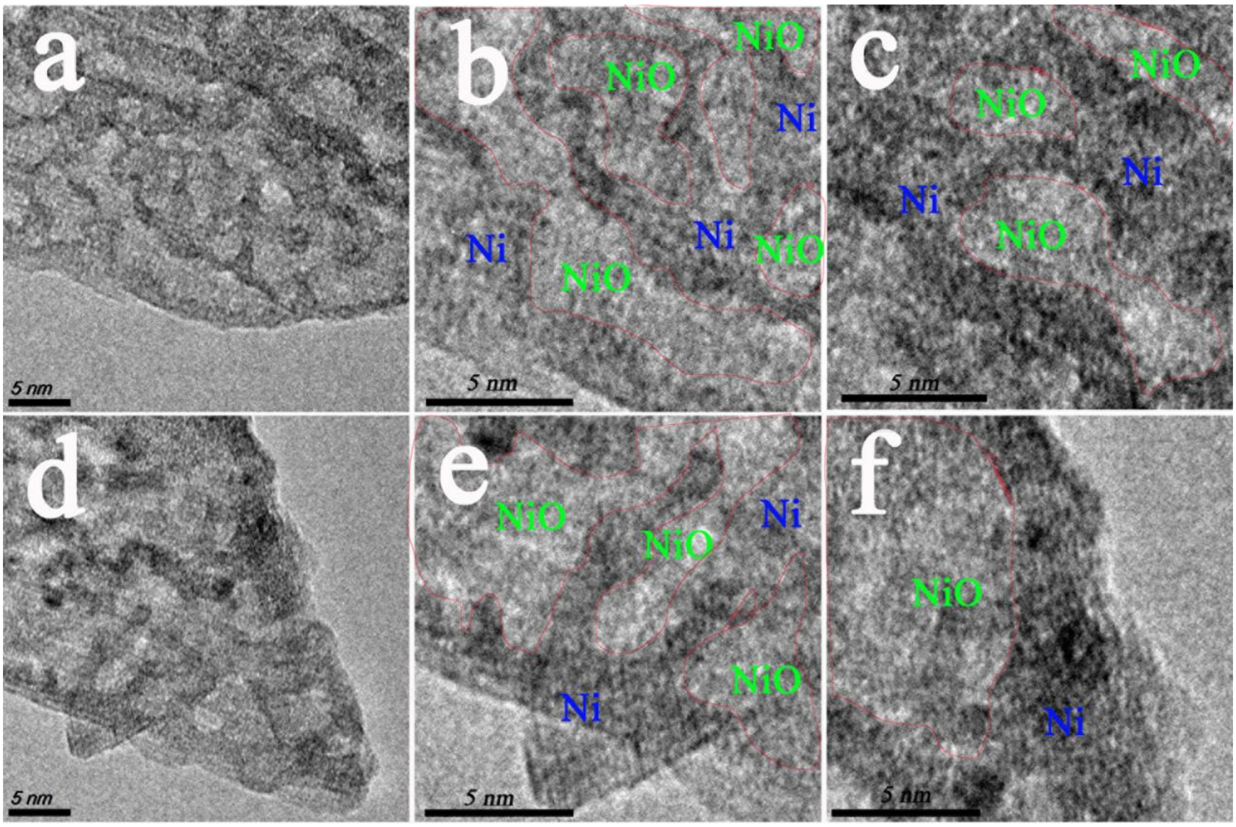

Fig. S13. HRTEM images of edge area from two different nanosheets in the Ni-NiO-5min samples.

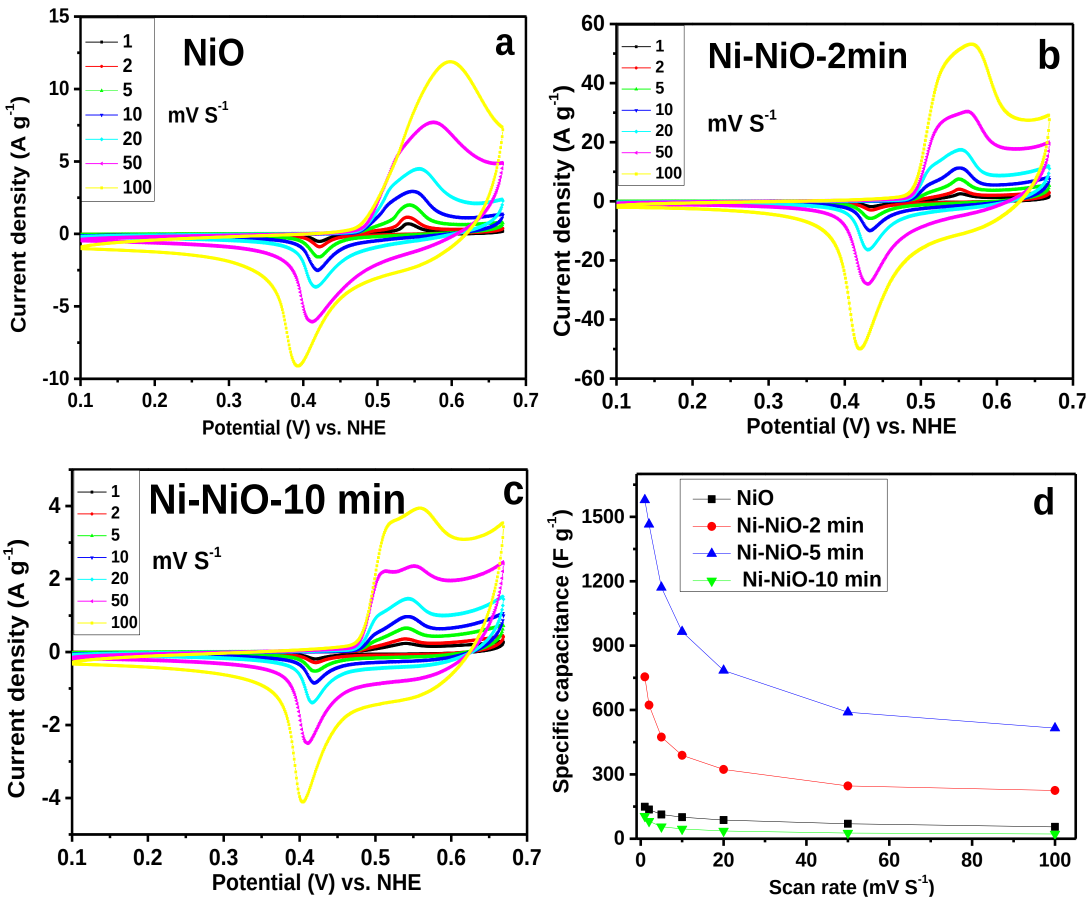

Fig. S14. CVs of different electrodes at different scan rates: (a) NiO; (b) Ni-NiO-2min; (c) Ni-NiO-10min; (d) specific capacitance versus CV scan rate for different NiO-based electrodes.

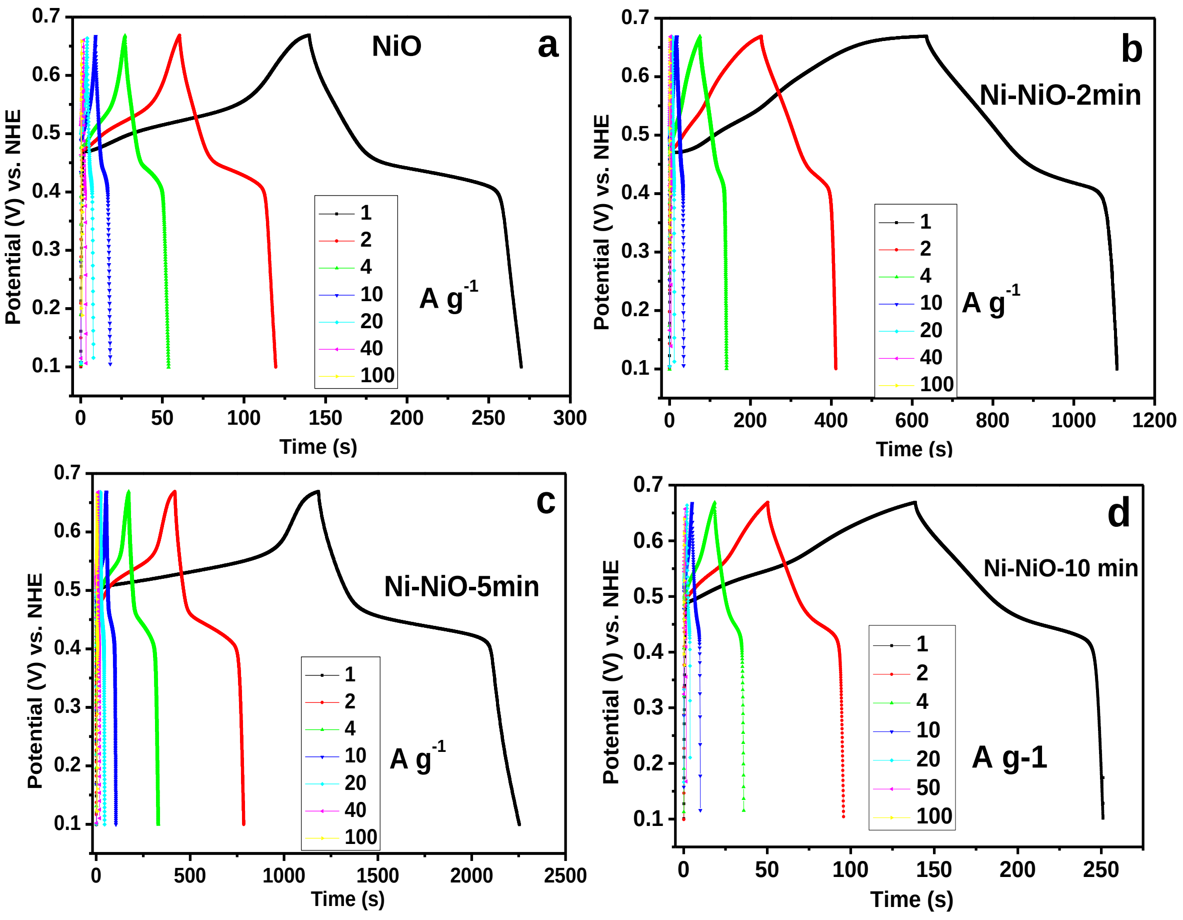

Fig. S15. Galvanostatic charge/discharge curves at different current densities: (a) NiO; (b) Ni-NiO-2min; (c) Ni-NiO-5min; (d) Ni-NiO-10min.

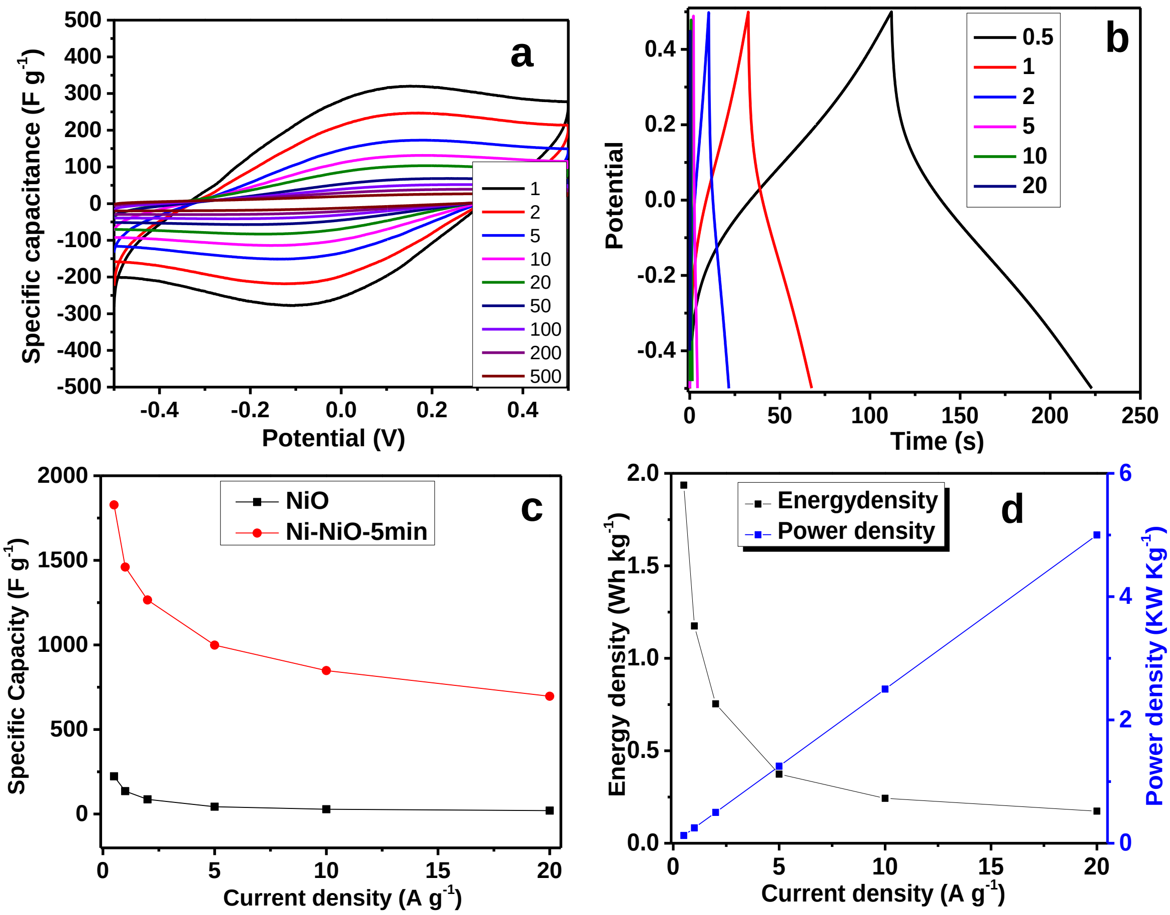

Fig. S16. (a) CVs of two-electrode cell based on pristine NiO at different scan rates; (b) galvanostatic charge/discharge curves at different current densities; (c) specific capacitance versus current density for NiO- and Ni-NiO-5min based symmetrical supercapacitors; and (d) energy and power densities versus current density for NiO symmetrical supercapacitor.

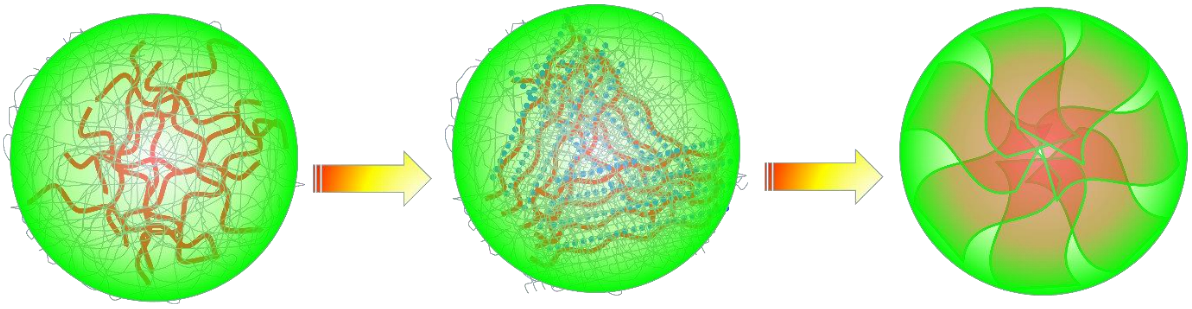

Fig. S17. Schematic of the formation mechanism of the Ni-NiO microflower.

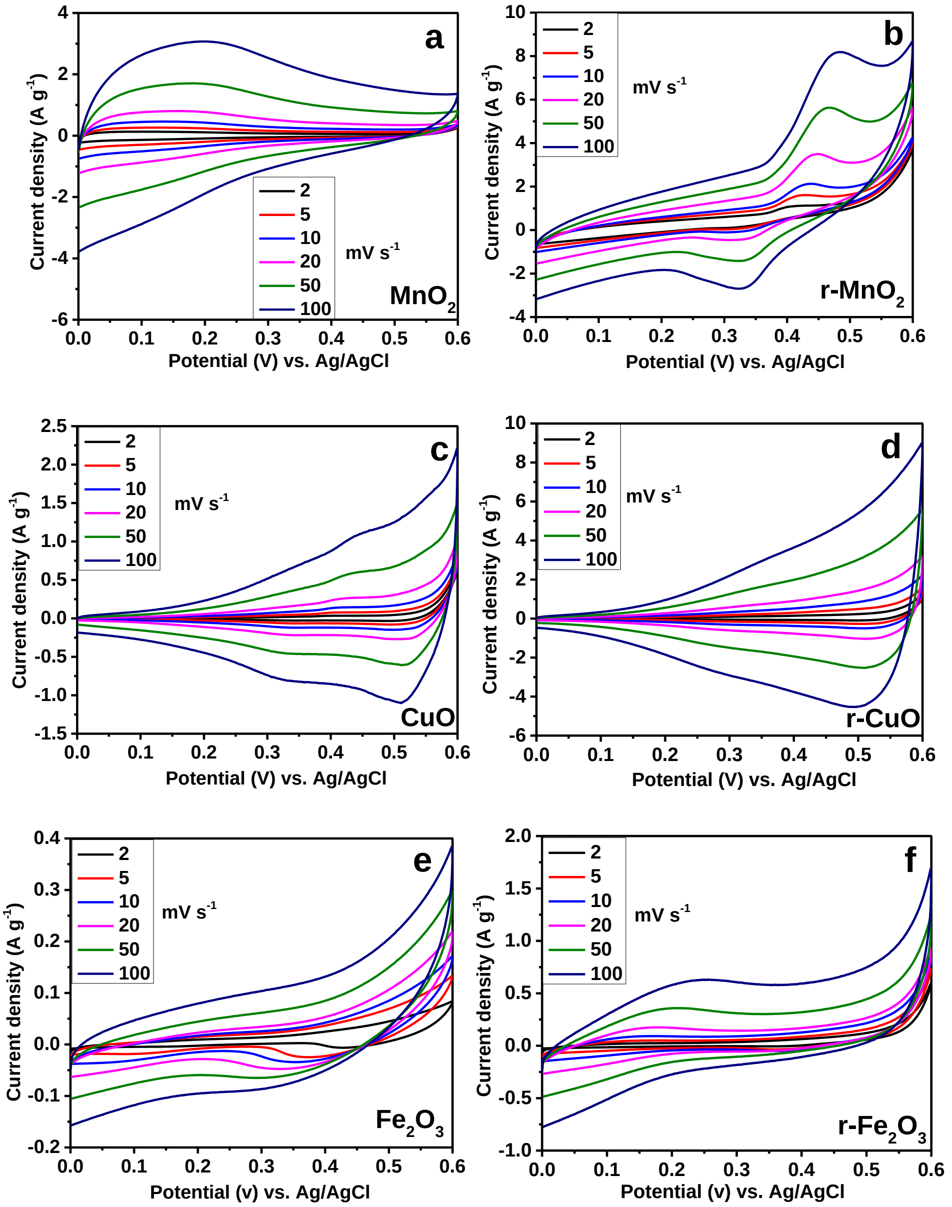

Fig. S18. CVs of different electrodes at different scan rates: (a) MnO_2; (b) *r*-MnO_2; (c) CuO; (d) *r*-CuO; (e) Fe_2O_3; (f) *r*- Fe_2O_3.

We also investigated the effect of metal oxides (such as r-CuO, r-MnO_2, and r-Fe_2O_3) on the supercapacitive behavior. CuO (Nanopowder, <50 nm) and MnO_2 (5 µm) were purchased from Sigma Aldrich, while Fe_2O_3 nanoparticles were synthesized in our lab. The three metal oxides were partially reduced using a similar procedure with H_2 as a reducing agent. They were initially heated to 350 ℃ at a rate of 5 ℃/min in argon and maintained at 350 ℃ with a flow of H_2/Ar (1:9) gas for 5 min. The electrochemical properties for these metal oxides were measured before and after reduction, as shown in Figs. S17 and S18. Compared with pure metal oxides, all the metal oxide after the H_2 reducing treatment showed an increase in capacity current or redox current, which is consistent with what we concluded in this work; i.e., partially reducing metal oxide could enhance the electrical conductivity and thus lead to a higher utilization of metal oxides.

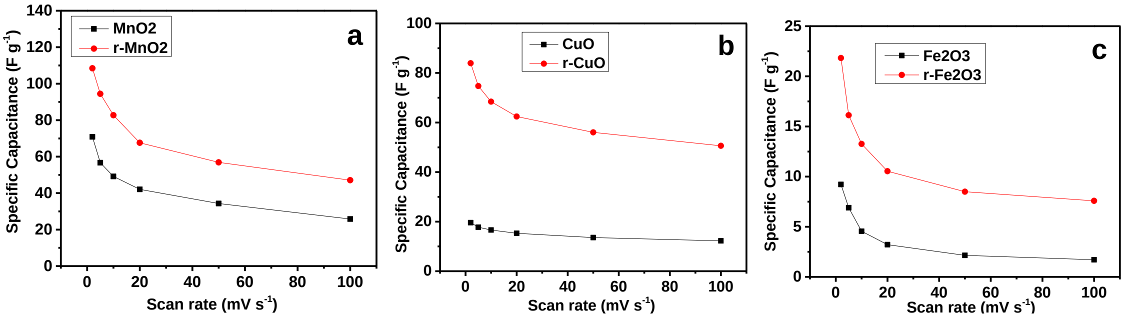

Fig. S19. Specific capacitance versus CV scan rate for different metal oxide electrodes, (a) MnO_2 and *r*-MnO_2; (b) CuO and *r*-CuO; (c) Fe_2O_3 and *r*- Fe_2O_3.

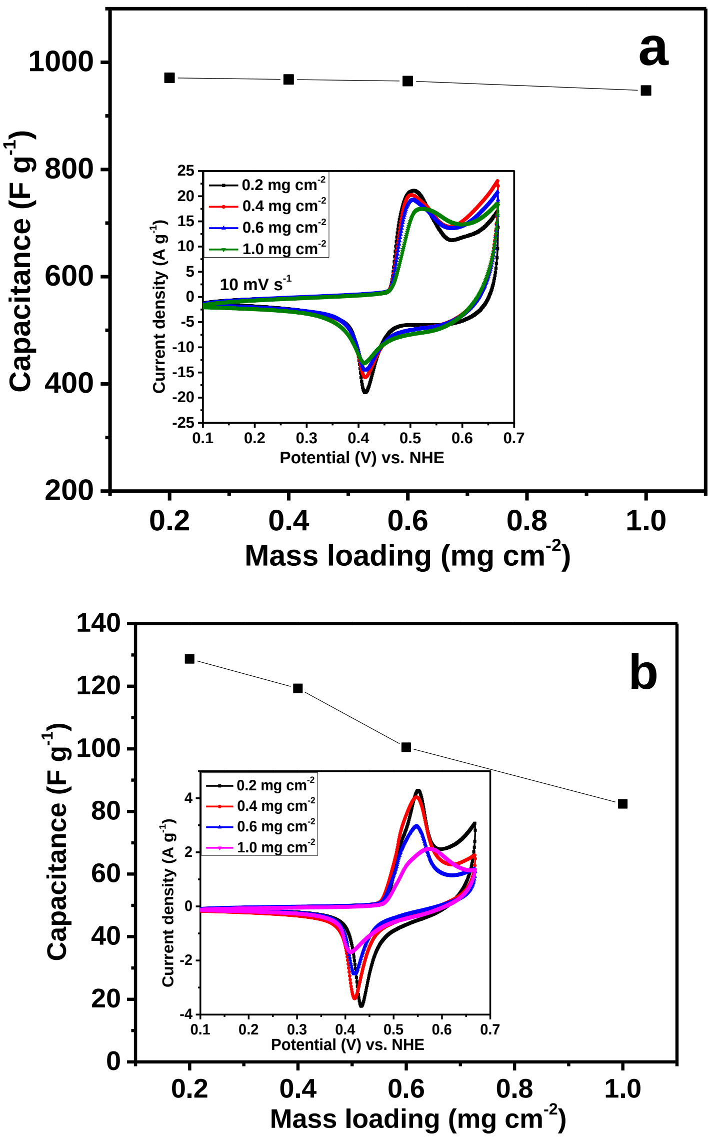

Fig. S20. The effect of mass loading of (a) Ni-NiO-5min and (b) NiO on specific capacitance values. Insets are the corresponding CVs of supercapacitors with different mass loadings at a scan rate of 10 mV s^{-1}.

The Ni-NiO-5mins electrodes show negligible decreases in specific capacitance values (from 971 F g^{-1} to 964 F g^{-1}) with increasing mass loadings from 0.2 mg cm^{-2} to 0.6 mg cm^{-2}, and the specific capacitance slightly decreases to 952.4 F g^{-1} when the mass loading increases to 1.0 mg cm^{-2}, indicating good utilization of the electrode material at a high mass loading. In contrast, the pristine NiO electrodes show a drastic decrease in specific capacitance from 128.7 to 82.4 F g^{-1} when mass loadings increase to 0.2 mg cm^{-2} and 1.0 mg cm^{-2}.

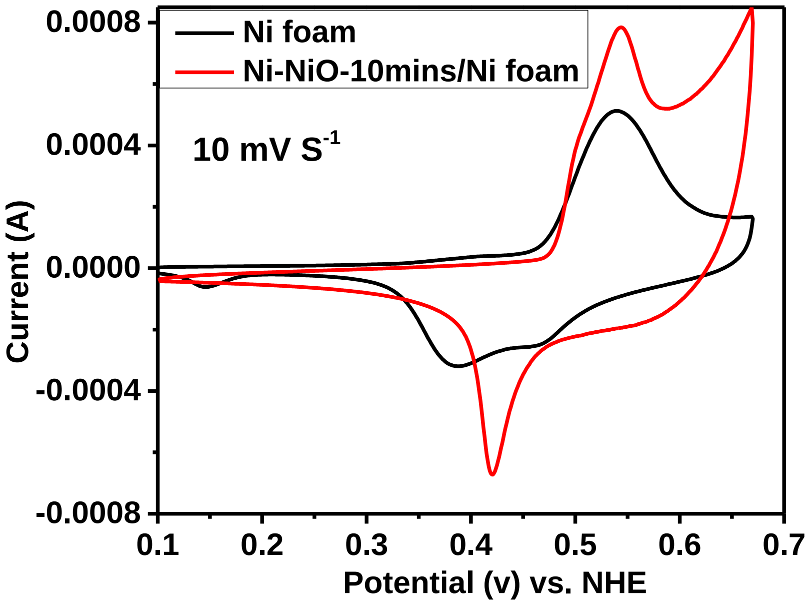

Fig. S21. CVs of bulk Ni foam and Ni-NiO-10min at a scan rate of 10 mV S^{-1}.

The bulk Ni foam current collector shows a slightly lower current than the **Ni-NiO-10min** sample (Fig. S20). Because the Ni-NiO-10min sample shows a smaller capacitance than pristine NiO, the capacitance contributed by the Ni foam is negligible for the Ni-NiO-5min sample.

The carbon cloth was purchased from Zoltek Company and was directly used as a current collector without pretreatment. The bare carbon cloth did show well-defined properties of an electric double-layer capacitor, as indicated by the rectangular CV at a high scan rate; however, the bare carbon cloth showed a capacitive current that is almost three orders of magnitude lower than the redox current of the Ni-NiO-5min-modified carbon cloth electrode. The calculated capacitance of the carbon cloth is less than 10 F g^{-1} at a scan rate of 10 mV s^{-1}.

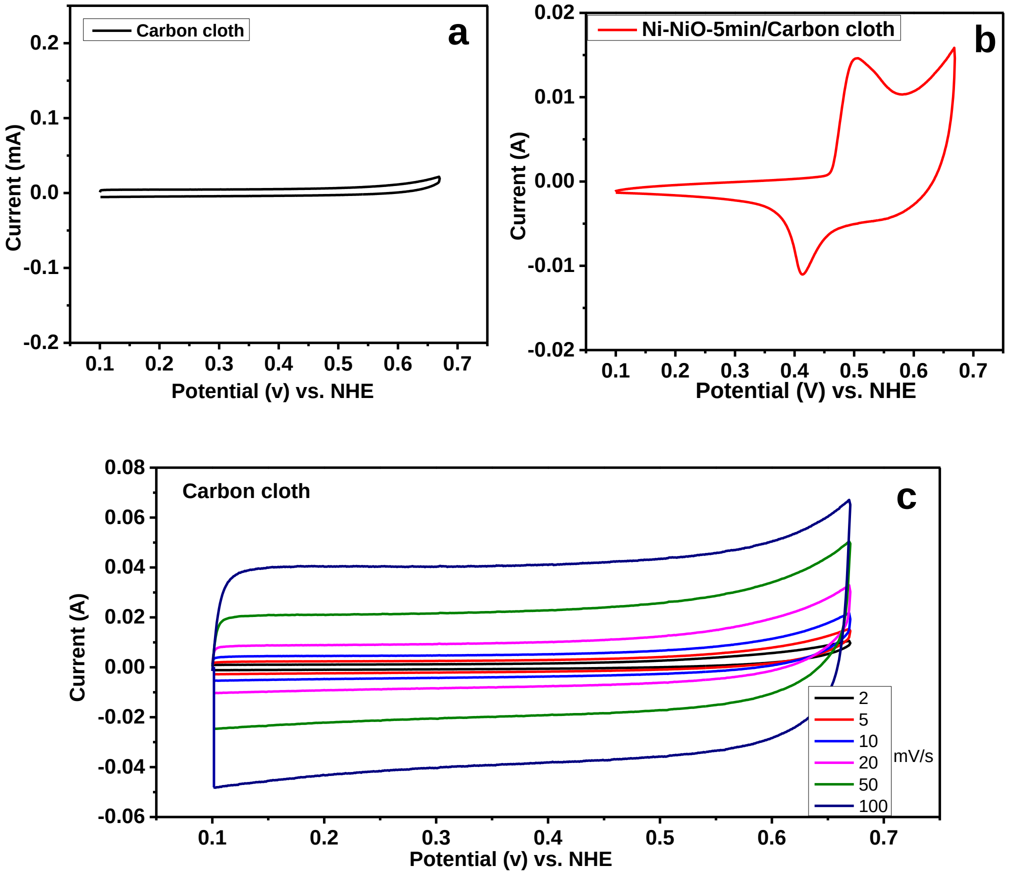

Fig. S22. CVs of (a) bare carbon cloth and (b) Ni-NiO-5min/carbon-cloth electrodes in 1 M KOH at a scan rate of 10 mV s^{-1} in a three-electrode system; (c) CVs of carbon cloth electrode in a three-electrode cell at different scan rates in 1 M KOH.

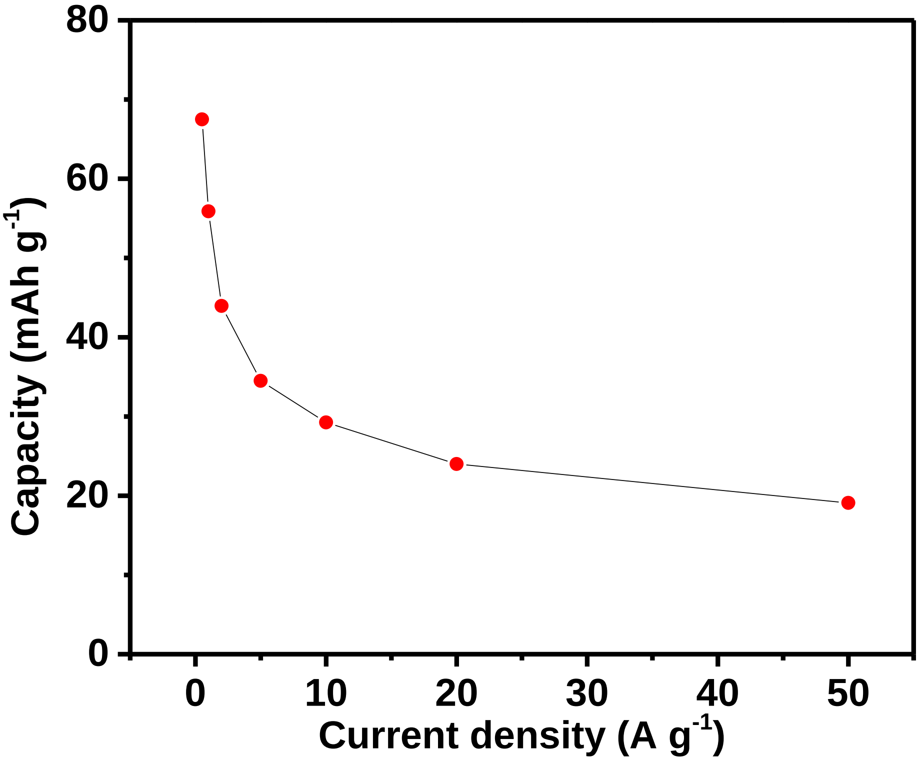

Figure S23. The capacity of the Ni-NiO-5min symmetrical capacitor cell at different current density.

Table S1. Performance comparison for NiO-based supercapacitors

NiO-based material	Specific capacitance, 3-electrode cell (F g^{-1})	Specific capacitance, 2-electrode cell (F g^{-1})	Volumetric specific capacitance (1 A g^{-1})	Ref.
Ni-NiO-5min microflower	1,998 (1 M KOH, 1 A g^{-1})	1,460 (1 M KOH, 1 A g^{-1})	1,352.7 F cm^{-3}	This work
NiO microflower	149.1 (1 M KOH, 1A g^{-1})	135.3 (1 M KOH, 1 A g^{-1})	100.1 F cm^{-3}	This work
NiO/CNT	1,329 (1 M KOH)	N/A	N/A	Ref [s2]
NiO microflower	265 (2 M KOH) 5 mV/s	N/A	N/A	Ref [s3]
1D NiO hollow nanostructures	702 (2.0 M KOH) 2 A g^{-1}	N/A	N/A	Ref [s4]
NiO nanoplatelet array	312 (1.0 M KOH)	N/A	N/A	Ref [s5]
NiO porous film on rGO sheet	432 (1.0 M KOH) 2 A/g	N/A	N/A	Ref [s6]
NiO–rGO	569 (1.0 M KOH) 2 A g^{-1}	N/A	N/A	Ref [s7]
3D nanoporous NiO film	1,800 (1.0 M KOH) 1 A g^{-1}	N/A	N/A	Ref [s8]
NiO nanoflakes	401, 0.5 mA cm^{-2}	N/A	N/A	Ref [s9]
NiO nanospheres	603, 0.5 A g^{-1}	N/A	N/A	Ref [s10]
NiO/Ni nanocomposites	905, 1 A g^{-1}	N/A	N/A	Ref [s11]
NiCo2O4 nanosheets	1,002, 1A g^{-1}	N/A	N/A	Ref [s12]
NiO-Go	375, 5 mV s^{-1}	N/A	N/A	Ref [s13]
NiO-TiO2 nanotube array	120 -300 (1 M KOH)	N/A	N/A	Ref [s14]

Note:

1. To our knowledge, there are few reports on using 2-electrode symmetrical cells to test NiO-based supercapacitors;

2. Both volumetric specific capacitance and energy density were calculated based on results with a 2-electrode symmetrical cell.

References for supporting information

[S1] Y. Korenblit, A. Kajdos, W. C. West, M. C. Smart, E. J. Brandon, A. Kvit, J. Jagiello, G. Yushin. Adv. Funct. Mater. 2012, 22, 1655–1662.

[S2] P. Lin, Q. She, B. Hong, X. Liu, Y. Shi, Z. Shi, M. Zheng and Q. Dong, J. Electrochem. Soc. 2010, 157, A818-A823.

[S3] Y. Ren and L. Gao, J. Am. Ceram. Soc., 2010, 93, 3560 - 3564.

[S4] G. Zhang, L. Yu, H. E. Hoster, X. W. Lou. Nanoscale, 2013, 5, 877.

[S5] J. Li, W. Zhao, F. Huang, A. Manivannan, N. Q. Wu, Nanoscale, 2011, 3, 5103-5109.

[S6] X. H. Xia, J. P. Tu, Y. J. Mai, R. Chen, X. L. Wang, C. D. Gu, X. B. Zhao, Chem. Eur. J. , 2011, 17, 10898-10905.

[S7] M. S. Wu, Y. P. Lin, C. H. Lin and J. T. Lee, J. Mater. Chem., 2012, 22, 2442-2448.

[S8] K. Liang, X. Tang, W. Hu., J. Mater. Chem., 2012, 22, 11062.

[S9] S. Vijayakumar, S. Nagamuthu, G. Muralidharan, ACS Appl. Mater. Interfaces 2013, 5, 2188-2196.

[S10] D. Han, P. Xu, X. Jing, J. Wang, P. Yang, Q. Shen, J. Liu, D. Song, Z. Gao, M. Zhang. J. Power Sources. 2013, 235 45-53.

[S11] Q. Lu, M. W. Lattanzi, Y. Chen, X. Kou, W. Li, X. Fan, K. M. Unruh, J. G. Chen, J. Q. Xiao. Angew. Chem. Int. Ed. 2011, 50, 6847-6850.

[S12] G. Zhang, X. W.) Lou, Sci Rep. 2013, 3, 1470.

[S13] H. Wang, C. M. B. Holt, Z. Li, X. Tan, B. S. Amirkhiz, Z. Xu, B. C. Olsen, T. Stephenson, D. Mitlin, Nano Res. 2012, 5, 605–617.

[S14] J. H. Kim, K. Zhu, Y. Yan, C. L. Perkins, A. J. Frank, Nano Lett. 2010, 10, 4099-4104.
